# Assessment of the Performance of Electrodialysis in the Removal of the Most Potent Odor-Active Compounds of Herring Milt Hydrolysate: Focus on Ion-Exchange Membrane Fouling and Water Dissociation as Limiting Process Conditions

**DOI:** 10.3390/membranes10060127

**Published:** 2020-06-20

**Authors:** Sarah Todeschini, Véronique Perreault, Charles Goulet, Mélanie Bouchard, Pascal Dubé, Yvan Boutin, Laurent Bazinet

**Affiliations:** 1 Department of Food Sciences, Université Laval, Québec, QC G1V 0A6, Canada; sarah.todeschini.1@ulaval.ca (S.T.); veronique.perreault.5@ulaval.ca (V.P.); 2 Laboratoire de Transformation Alimentaire et Procédés ElectroMembranaires (LTAPEM, Laboratory of Food Processing and ElectroMembrane Processes), Université Laval, Québec, QC G1V 0A6, Canada; 3 Institute of Nutrition and Functional Foods (INAF), Université Laval, Québec, QC G1V 0A6, Canada; yvan.boutin@tbt.qc.ca; 4 Department of Phytology, Université Laval, Québec, QC G1V 0A6, Canada; charles.goulet@fsaa.ulaval.ca; 5 Investissement Québec-Centre de Recherche Industrielle du Québec (CRIQ, Quebec Investment–Industrial Research Center of Quebec), Québec, QC G1P 4C7, Canada; melanie.bouchard@invest-quebec.com (M.B.); pascal.dube@invest-quebec.com (P.D.); 6 Centre Collégial de Transfert de Technologie en Biotechnologie (TransBIOTech, College Center for Technology Transfer in Biotechnology), Lévis, QC G6V 6Z9, Canada

**Keywords:** electrodialysis, deaerator, herring milt hydrolysate, deodorization, off-flavors, trimethylamine, fouling, water dissociation

## Abstract

Herring milt hydrolysate (HMH), like many fish products, presents the drawback to be associated with off-flavors. As odor is an important criterion, an effective deodorization method targeting the volatile compounds responsible for off-flavors needs to be developed. The potential of electrodialysis (ED) to remove the 15 volatile compounds identified, in the first part of this work, for their main contribution to the odor of HMH, as well as trimethylamine, dimethylamine and trimethylamine oxide, was assessed by testing the impact of both hydrolysate pH (4 and 7) and current conditions (no current vs. current applied). The ED performance was compared with that of a deaerator by assessing three hydrolysate pH values (4, 7 and 10). The initial pH of HMH had a huge impact on the targeted compounds, while ED had no effect. The fouling formation, resulting from electrostatic and hydrophobic interactions between HMH constituents and ion-exchange membranes (IEM); the occurrence of water dissociation on IEM interfaces, due to the reaching of the limiting current density; and the presence of water dissociation catalyzers were considered as the major limiting process conditions. The deaerator treatment on hydrolysate at pH 7 and its alkalization until pH 10 led to the best removal of odorant compounds.

## 1. Introduction

Due to the important increase in fish consumption, by-products generated by industries have known an important increase over the last twenty years [1]. One major way of valorizating these by-products is based on their hydrolysis through the use of chemicals or enzymes. The resulting hydrolysates have gained more and more attention from the food, nutraceutical and cosmetics sectors, since they could be of biological interest, having antioxidant, anticancer, antidiabetic, antihypertensive or antimicrobial activities [2,3,4,5,6,7]. Recently, the valorization of herring milt hydrolysate (HMH) has been much more considered, since it contains high value-added molecules, such as peptides, amino acids, nucleic acids and vitamins, and since it has already proven its relevance regarding metabolic syndrome illnesses [1,8]. However, in spite of these promising aspects, HMH, similarly to other fish hydrolysates, presents the substantial drawback of being associated with unpleasant smells [9]. Since odor is an important criterion for consumer acceptance of a product, there is a real need to overcome this main issue [10].

Unpleasant odors in fishery products and by-products such as hydrolysates can be, in general, ascribed to the contribution of several compounds [9,11]. Nevertheless, all these compounds present the similarities of being volatile, low-molecular-weight and hydrophobic molecules [12]. Among these compounds, trimethylamine (TMA), a tertiary amine characterized by a strong fishy odor, is the most well-known agent involved in the off-flavors of marine products [13]. With other amines like dimethylamine (DMA), TMA is considered to be a major indicator of fish spoilage [14]. TMA and DMA originate from the breakdown of trimethylamine oxide (TMAO) due to bacterial and enzymatic reactions, respectively, as well as environmental conditions [15]. In addition to nitrogen-containing compounds, other volatile compounds belonging to various chemical groups, such as aldehydes, ketones or alcohols, have already been proven to be associated with off-flavors in fishery materials [9,11,16]. These latter compounds originate from enzymatic reactions, lipid oxidation, microbial actions and environmental or thermic stresses [10]. Nonetheless, as no study has been carried out so far regarding the odor of HMH, there is no evidence concerning the contribution of these compounds in this type of matrix, and this needs to be further investigated. 

Different strategies are currently available to remove off-flavors. Depending on their principles, they can be divided into three groups of deodorization methods: biological, chemical and physical methods. Firstly, biological methods are based on the use of microorganisms. However, their action spectrum is often reduced, as they target specific compounds, and their mechanisms remain inaccurate [9,17,18]. Chemical methods involve processes like ozonation or the use of antioxidants. The ozonation process presents the main drawback of giving rise to side reactions [19], while the use of antioxidants would be more considered as a preventive measure than a curative one [9,20]. Finally, physical methods include various processes, such as extraction or adsorption on microporous materials. Extraction methods can involve high temperatures that may damage thermolabile compounds [21], while the action of adsorption methods is limited [9]. Thus, disadvantages seem to be associated with all the available methods. Surprisingly, other physical processes, based on the use of selective membranes such as pressure-driven and electromembrane processes, have never been investigated for deodorization purposes so far. Therefore, developing a new deodorization method based on the use of membranes would be an innovative and challenging idea. 

It is of importance to note that pressure-driven processes have already been reported for the recovery of valuable components from cooking effluents of seafood processing industries. In that case, pressure-driven technologies allowed to obtain two distinct fractions: one rich in low-sized compounds, including volatile compounds, and another one containing purified water [22,23,24]. Nevertheless, in a deodorization context, the aim is only to isolate volatile compounds. Therefore, the use of pressure-driven technologies does not look appropriate for this problem. Concerning electromembrane processes, despite the fact they have never been used for deodorization purposes, the impact of a demineralization process performed by conventional electrodialysis (ED) on volatile compounds was already considered by Cros et al. (2005) and Chindapan et al. (2011). Interestingly, these studies showed that ED could lead to a decrease in the odor intensity of the treated product resulting from a decrease in the abundance of certain volatile compounds [25,26]. More specifically, Cros et al. (2005) observed a decrease in (Z)-4-heptenal, 2,3-butanedione, 3-octen-2-one, limonene, phenol and 1-propanol, while Chindapan et al. (2011) experienced a decrease in TMA, 2,6-dimethylpyrazine, various carboxylic acids and phenol, as well [25,26]. According to these results, ED would be a plausible effective process to lower the concentration of these specific molecules. To assess the feasibility of ED on HMH, it appears to be even more necessary to identify the volatile compounds contributing to its odor. Furthermore, since ED is based on the selective migration of ionic species through ion-exchange membranes (IEM) using an electric field as driving force [27], it is particularly interesting to mention that two of the major compounds associated with off-flavors in seafood products, TMA and DMA, are positively charged when the pH is lower than their pKa values, respectively equal to 9.80 and 10.70 [28], making these cations likely to migrate during an ED process. 

In this context, the aim of the present study was to (1) identify the main volatile compounds of HMH contributing to its odor, (2) challenge ED as a new potential deodorization method by comparison with a deaerator and (3) understand the potential fouling mechanisms. Even if the purpose of a deaerator is more to remove air from liquid samples, as compounds responsible for off-odors are volatile, they are removed at the same time as gases, making this device suitable as a control for this type of problem.

## 2. Materials and Methods

### 2.1. Materials

#### 2.1.1. Chemicals

Sodium sulphate (Na_2_SO_4_), potassium chloride (KCl), sodium chloride (NaCl), methylene chloride and nonyl acetate were purchased from Fisher (Montréal, QC, Canada). Hydrochloric acid (HCl) and sodium hydroxide (NaOH) were obtained from VWR (Montréal, QC, Canada). TMAO, TMA and DMA standards came from Sigma-Aldrich (St-Louis, MO, USA). Helium and nitrogen were obtained from Praxair (Mississauga, ON, Canada). Oxygerm, Blizzard and Extrem solutions were purchased from Sani Marc (Victoriaville, QC, Canada). 

#### 2.1.2. HMH

HMH powder, the composition of which is listed on Table 1, was provided by Ocean NutraSciences (Matane, QC, Canada). The hydrolysate was stored at −30 °C under vacuum and protected from light before its use.

### 2.2. Methods

#### 2.2.1. Protocols

Two protocols were specifically designed for this study. The first aimed to identify the volatile compounds of the HMH and their contribution to its odor, while the second assessed the potential of ED to remove these compounds.

##### Volatile Compounds Analysis

Volatile Compound Extraction

Volatile compounds were extracted according to the procedure described by Tremblay et al. (2020) [29]. Briefly, 6 g of solid samples dissolved in 24 mL of deionized water were absorbed by cotton balls. The soaked cotton balls were then introduced into glass tubes in which air circulated for 1h at room temperature. Volatile compounds were collected on a divinylbenzene column (HayeSep^®^ Q 80/100, Bandera, TX, USA). After that, they were eluted with 150 µL of an elution solvent. The elution solvent consisted in dichloromethane containing nonyl acetate as an internal standard (3.71 × 10^−5^ M). The extracts were stored at −80 °C before undergoing further analyses.

Volatile Compound Identification

Volatile compounds were identified using a gas chromatography-mass spectrometry (GC-MS) system consisting of a 7890B GC (Agilent Technologies, Santa Clara, CA, USA) equipped with a 5977B mass selective detector (MSD) with a high efficiency source (Agilent Technologies, Santa Clara, CA, USA). The extract (1.8 µL) was injected in splitless mode into a capillary column (DB-5MS Ultra Inert, 30 m length × 250 µm id, 1 µm thickness) (Agilent Technologies, Santa Clara, CA, USA). The flow rate of the carrier gas (helium) was 1.3 mL/min. The temperature of the oven was programmed according to the following steps: from 35 °C (temperature hold for 1.46 min) to 47 °C at 6 °C/min, and to 250 °C (temperature hold for 3 min) at 10 °C/min. MSD conditions were as follows: source temperature, 230 °C; quad temperature, 150 °C; ionization energy, 70 eV; mass range, 30 to 250 arbitrary unit of mass (a.m.u); scan rate, 6,1 scans/s. Data obtained were processed with MassHunter Qualitative Analysis software version B.07.00 (Agilent Technologies, Santa Clara, CA, USA). Volatile compounds were identified by comparing their mass spectra with those available in the NIST 17.L mass spectral database (National Institute of Standards and Technology, Gaithersburg, MD, USA), and were reported when the match degree exceeded 60%.

Most Potent Odor-Active Volatile Compound Determination

The most potent odor-active volatile compounds of the HMH were determined using gas chromatography-olfactometry (GC-O) and the frequency detection method. The GC-O system consisted of a 7890B GC (Agilent Technologies, Santa Clara, CA, USA) equipped with a 5977B MSD with a high efficiency source (Agilent Technologies, Santa Clara, CA, USA) and an olfactory detection port ODP3 (Gerstel, Linthicum, MD, USA) supplied with humidified air to avoid the drying of the mucous membrane in the nasal cavity. The GC effluent was split in the ratio 1: 2 between the MSD and the olfactory detection port. The extract (1.8 µL) was analyzed according to the same procedure as those described previously. A panel made of 13 judges (11 females and 2 males between 23 and 50 years old) took part in the olfactometric experiment. They all previously followed a training session to become familiar with the GC-O system. The sniffing of the HMH extract took place during a session of 26.76 min. The judges were asked to assess the intensity of each odorant on a scale of 1–4 (1 = very weak odor intensity; 4 = very strong odor intensity) and, at the same time, to qualify the perceived odor as bad, good or ok (the latter being if the perceived odor was acceptable without being all that pleasant). Data obtained from GC-MS were processed with MassHunter Qualitative Analysis software version B.07.00 (Agilent Technologies, Santa Clara, CA, USA), while those obtained from olfactometry were treated by Gerstel ODP dataviewer software version 1.0.2.8 (Linthicum, MD, USA). Compounds detected by at least 5 of the 13 judges were considered as the most potent odor-active volatile compounds.

Analysis of the TMAO, TMA and DMA Contents

As the GC-MS method previously described was not sensitive enough to detect TMAO, TMA and DMA, another procedure was used. Samples were prepared as follows: 2 mL standards or samples pipetted into 20-mL vials were alkalized to pH 10 using 0.01, 0.1 or 5 M NaOH. The molarity of the NaOH solution used for this purpose depended on the initial pH of the analyzed samples in order to avoid any dilution effect. TMAO, TMA and DMA contents were then determined using headspace (HS) gas chromatography equipped with a nitrogen-phosphorus detector (NPD). Briefly, samples were equilibrated for 10 min in a 7697A headspace sampler (Agilent Technologies, Santa Clara, CA, USA) at 90 °C. Then 1-mL sample was injected into a 7890B GC (Agilent Technologies, Santa Clara, CA, USA) equipped with a NPD. The loop and transfer line were at 110 °C and 115 °C, respectively. Volatile amines were separated using a capillary column (CP-Volamine, 30 m length x 0.32 mm id) (Agilent Technologies, Santa Clara, CA, USA). GC conditions were as follows: split injection, 1:5; injector temperature, 250 °C; helium carrier gas flow, 3 mL/min; detector temperature, 300 °C; oven programmed from 50 °C (4 min) to 200 °C at 25 °C/min. Data were processed with Open Lab CDS EZChrom software version A.03.02.023 (Agilent Technologies, Santa Clara, CA, USA).

##### Deodorization by ED

ED Cell and Configuration

**Figure 1:**
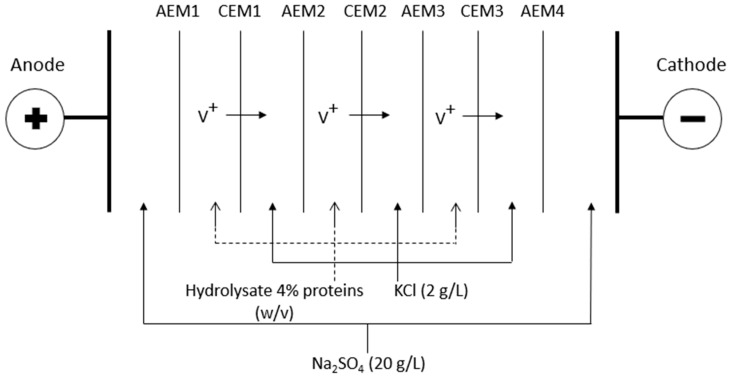
Electrodialysis cell configuration; V^+^: cationic volatile compounds, AEM: anion-exchange membrane, CEM: cation-exchange membrane.

The ED cell used was a MP type cell with an effective area of 100 cm^2^, manufactured by Electrocell AB (Taby, Sweden), including 4 Neosepta AMX-FG anionic membranes (AEM, Astom Ltd., Tokyo, Japan) and 3 Neosepta CMX-FG cationic membranes (CEM, Astom Ltd., Tokyo, Japan) (Figure 1). This arrangement defined three closed loops containing the 20 g/L Na_2_SO_4_ electrolyte solution (3 L), the 2 g/L KCl solution (3 L) for the potential recovery of volatile compounds and the dry HMH dissolved to 4% of proteins in water overnight at 4 °C under nitrogen, protected from light; the initial pH of 7.3 was adjusted to the desired value (3 L). Each loop was connected to an external tank allowing a continuous recirculation of the solutions. Each tank was closed in order to avoid any loss of volatile compounds due to their volatility. All the solutions were circulated at a flow rate of 3 L/min (1.5 psi) using three centrifugal pumps. The cathode and anode used were respectively a 316 stainless steel electrode and a dimensionally-stable electrode (DSA-O_2_). A 0-100 V power supply was employed to generate the potential difference between the electrodes.

ED Parameters

The ED experiment was conducted for 240 min. This treatment duration was set in order to meet two main requirements: allowing the potential migration of volatile compounds without risking the degradation of polyunsaturated fatty acids present in the HMH. Also, for these reasons, in addition to microbiological concerns, temperature was controlled around 10 °C during all the ED treatments.

ED treatments were performed under different constant voltage conditions corresponding to no current application (a residual electrode potential difference of 0.7 V) and current application (voltage value of 10 V) to assess the impact of current on the volatile compound content of the HMH. Also, to assess if TMA and DMA were able to migrate in their cationic forms, the ED treatments were conducted on HMH solution preliminarily acidified at pH 4 or pH 7 with, respectively, 14 and 7 mL of 6 M HCl. Indeed, while pH is inferior to the pKa values of these two amines, namely 9.80 for TMA and 10.70 for DMA [28], they became positively charged. The pH of hydrolysate was maintained either at 4 or at 7 with, respectively, a total addition of 25 and 10 mL of 6 M HCl during the ED run. 

45-mL samples of hydrolysate and KCl recovery solutions were collected at the initial and final times. These samples were stored at −30 °C and protected from light before being further analyzed in terms of volatile compounds and ash contents. Each condition was conducted in triplicate and in a random order. Following each run, the ED cell was rinsed with NaCl 2% (w/v) twice. The stack was cleaned with 0.1 N HCl and 0.1 N NaOH solutions every four runs. All the membranes were characterized in terms of average thickness and conductivity before any experiment and then after each run. Different membranes were used for the different conditions. However, the same membranes were used for the three replicates of the same condition. 

Comparison with a Deaerator

To assess the performance of ED as a new potential deodorization method, deaerator assays were carried out. The device used was a deaerator model ERV2 (Koruma, Neuenburg, Germany). Based on preliminary tests, HMH (3L) was treated for 30 min in closed circuit under 100 Torr. In this case, treatments were performed on hydrolysate solution prepared according to the same procedure as those for ED runs. The only difference was that, in addition to pH 4 and 7, experiments on hydrolysate solution alkalized to pH 10 with 6 M NaOH were conducted as well. Indeed, in the case of deaerator, it did not matter to charge TMA and DMA molecules, as this device only uses the volatility state of compounds. Similarly to the ED experiments, 45-mL samples of hydrolysate were collected at the initial and final time. They were then stored at −30 °C and protected from light before being further analyzed in terms of volatile compounds. Experiments were conducted in triplicate for each studied pH value. Before use, the deaerator was decontaminated with Oxygerm solution, involving a complex blend of organic acids and powerful oxidizers. After each run, the deaerator was cleaned with Blizzard and Extrem solutions that are, respectively, a foaming chlorinated alkaline degreaser and a blend of wetting, sequestering agents and caustic soda.

#### 2.2.2. Analyses

##### pH

The pH of the HMH and KCl recovery solutions was measured using a pH-meter model SP20 (Thermo Orion, West Chester, PA, USA) equipped with a VWR Symphony epoxy gel combination pH electrode (Montreal, QC, Canada). During the first hour of the ED run, pH values were recorded every 5 min and then every 15 min. 

##### Conductivity

The conductivity of hydrolysate and KCl recovery solutions was measured using a YSI conductivity meter (Model 3100) equipped with a YSI immersion probe model 3252, (cell constant K = 1 cm^−1^) (Yellow Springs Instrument Co., Yellow Springs, OH, United States). Similarly to the pH measurements, the conductivity values were recorded every 5 min during the first hour of the ED run, and then every 15 min. The demineralization rate (DR, in %) of the HMH and the mineralization rate (MR, in %) of the corresponding KCl recovery solution were determined according to Equations (1) and (2), respectively, where *k*_t_ refers to the solution conductivity at time t, and *k*_0_ refers to the initial solution conductivity [30].

(1) $$DR=\left(1-\frac{k_{t}}{k_{0}}\right)\times \;100$$

(2) $$MR=\left(1-\frac{k_{0}}{k_{t}}\right)\times \;100$$

##### Ash Content

Ash content (in %) was determined using a method adapted from the Association of Official Analytical Chemists [31]. Briefly, 10 mL samples of HMH and KCl recovery solutions at initial and final times were weighted before being dried overnight at 105 °C in an oven (VWR Gravity Convection Oven, Radnor, PA, USA). Dried samples were then reduced to ashes in a furnace at 550 °C until they turned white. Samples were weighted after cooling, and the ash content was determined according to Equation (3), where m refers to the measured weight.

(3) $$Ash\;content=\left(\frac{m_{crucible+ashes}-m_{crucible}}{m_{crucible+sample}-m_{crucible}}\right)\times \;100$$

The DR (in %) and the MR (in %) were also determined based on the ash content values according to Equations (4) and (5).

(4) $$DR=\left(1-\frac{ash\;content\;at\;time\;t}{ash\;content\;at\;initial\;time}\right)\times 100$$

(5) $$MR=\left(1-\frac{ash\;content\;at\;initial\;time}{ash\;content\;at\;time\;t}\right)\times 100$$

##### Global System Resistance

The global system resistance (R, in Ω) was calculated based on the Ohm’s law (R = U/I). As voltage (U, in V) value was maintained constant during all of the ED runs, only current intensity (I, in A) values were recorded, which were directly obtained from the power supply. 

##### Membrane Thickness

Membrane thickness was measured before and after each ED run in 0.5 M NaCl solution, according to the same procedure as Lemay et al. (2019) [32], using an electronic digital micrometer equipped with a 10-mm-diameter flat contact point from Marathon watch company LTD (Richmond Hill, ON, Canada). Six measurements, taking place at different locations on the membrane’s surface, were used to obtain the average membrane thickness.

##### Membrane Electrical Conductivity

Membrane electrical conductivity was measured before and after each ED run in 0.5 M NaCl solution, according to the procedure described by Lemay et al. (2019) [32], using a YSI conductivity meter model 3100 (Yellow Springs Instrument Co., Yellow Springs, OH, USA) equipped with a specially designed clip from the Laboratoire des Matériaux Echangeurs d’Ions (Université Paris XII, Créteil, Val de Marne, France). 

##### Volatile Compound Content

Volatile compounds determined as being the most potent odor-active compounds of the studied HMH, according to the GC-O procedure described previously, were used as deodorization indicators. Volatile compounds of HMH and KCl recovery solutions at initial and final times were obtained and analyzed according to the same extraction and GC-MS procedures described previously. The only difference was that extraction was performed on 45-mL liquid samples instead of solid samples. The area under each peak corresponding to the most potent odor-active volatile compounds was considered to assess their abundance in the different samples. 

The TMAO, TMA and DMA contents of HMH and KCl recovery solutions were also determined according to the procedure described previously. The quantification was carried out with a calibration curve of known amounts of TMAO, TMA and DMA standards (from 25 ppm to 755 ppm for TMAO; from 2.5 ppm to 10 ppm for TMA and DMA). 

##### Statistical Analyses

Analyses of variance (ANOVA) were performed, using SAS software version 9.4 for Windows, on data concerning ED parameters, as well as those regarding volatile compounds (SAS Institute Inc., Cary, NC, USA). A Tukey test (α = 0.05 as probability level) was used to compare the different treatments.

## 3. Results and Discussion

### 3.1. Volatile Compound Analysis

#### 3.1.1. Overall Content in Volatile Compounds

The overall content of the HMH was determined using GC-MS, while HS-GC-NPD was used to verify the presence of TMAO, TMA and DMA. The GC-MS procedure led to the identification of a total of 86 compounds, as listed in Table 2. Although a minimal match degree of 60% was initially selected, all of the volatile compounds were identified with a score ranging from 72.29% to 98.7%. The identified volatile compounds mainly belonged to eight groups, based on their chemical structures. More specifically, volatile compounds included 27 aldehydes, 17 ketones, 12 alcohols, 8 alkenes, 8 nitrogenous compounds, 5 alkanes, 2 furans and 2 esters, while the 5 other compounds were miscellaneous representatives of other chemical groups. Aldehydes and ketones were the most abundant compounds of the hydrolysate. Aldehyde compounds included saturated and unsaturated compounds. The unsaturated aldehydes involved alkenals, alkadienals and aromatic aldehydes. Aldehyde compounds generally originate from lipid oxidation [11,33]. This would be consistent with the composition of HMH, as it results from a rich source of polyunsaturated fatty acids, as shown in Table 1. Aldehydes can also derive from the Strecker degradation of amino acids [34]. Strecker degradation refers to the reaction involving a dicarbonylated compound and an amino acid [35]. Concerning the ketones content, it may also be the result of lipid and/or amino acid degradation [34]. Alkanes, alkenes and alcohols, also identified in large numbers in the HMH, are mainly known to directly derive from lipid oxidation [35,36,37]. Then, among the nitrogenous compounds content, 8 compounds were identified by GC-MS, while TMAO, TMA and DMA, also listed in Table 2, were detected by HS-GC-NPD. On the one hand, among the nitrogenous compounds identified by GC-MS, 2 pyrazines and 2 thiazoles were detected. More precisely, concerning the pyrazine content, methylpyrazine and 2,5-dimethylpyrazine were identified. Pyrazines are generally formed during Maillard or pyrolysis reactions in heat-processed foods [36,38]. However, despite the fact that high temperatures promote pyrazine formation, mild temperatures are also sufficient to lead to the formation of these compounds [13]. In the present study, since the HMH was obtained through enzymatic hydrolysis under minor heating, followed by a drying step by atomization involving higher temperatures, the two pyrazines identified might have been formed during one of these steps. Pyrazines can be biosynthesized by microorganisms, as well [34,36]. The thiazole content of HMH involved 2 compounds, 2-acetylthiazole and benzothiazole. Thiazole are sulfur-containing compounds that are supposed to originate from Strecker degradation [35]. Surprisingly, while Cha and Cadwaller (1995a) studied the volatile components of various fish and crustacean pastes, including herring paste and shrimp pastes, they identified 2-acetylthiazole only in shrimp paste, while benzothiazole was only identified in anchovy and hair tailed viscera pastes. Nonetheless, they were not detected in herring paste [39]. Regarding the four other nitrogenous compounds identified by GC-MS, three of them, namely 1-methyl-1H-tetrazole, 3-methyl-butanenitrile and 3-methyl oxime butanal, were not evidenced in fishery materials. These compounds may be characteristic of the volatile content of HMH. However, N-nitrosodimethylamine was already known to be generated from TMA and DMA [40]. On the other hand, the HS-GC-NPD procedure allowed the detection of TMAO, TMA and DMA (Table 2). In general, these compounds play a key role in the volatile content of fishery products. TMAO is a well-known osmoregulator, mainly found in marine fish, which aims to counteract protein destabilization [14]. The transformation of TMAO leads to the formation of TMA and DMA. More specifically, TMA can be formed from TMAO through bacterial degradation, while the formation of DMA is mainly attributed to the action of the endogenous TMAO aldolase enzyme [14,41]. Moreover, while TMAO is in presence of reducing agents, these molecules would be generated as well. Finally, compounds belonging to furan and ester groups were also found in HMH. More specifically, two furan compounds, 2-methylfuran and 5-isopropyl-3,3-dimethyl-2-methylene-2,3-dihydrofuran, were identified. Furans are known to originate from lipid oxidation [35]. Another mechanism of the formation of furan compounds involves Maillard and Strecker reactions [38]. Concerning the ester compounds identified, cis-cyclohexane-1,4-dimethanol diacetate and 2,2,4-trimethyl-1,3-pentanediol diisobutyrate, they may have originated from the esterification reaction occurring between alcohols and carboxylic acids generated through microbial or enzymatic lipid degradation [39]. It is of interest to note that Cha and Cadwallader (1995a) identified several ester compounds in herring paste, but none of them corresponded to those identified in this study. In addition, in herring paste, a high content of esters was observed. This should probably be linked to the fermentation process taking place in this type of products [39]. Also, it is interesting to note that, in this study, no carboxylic acid was identified. Whilst this was consistent with the study of Cha and Cadwaller (1995a) dealing with herring paste, this was not in line with the study of Aro et al. (2002), in which few carboxylic acids were identified in herring raw material [39,42]. Carboxylic acids are known to be lipid oxidation products, or to originate from amino acid degradation [42]. The absence of carboxylic acids might be a property of the volatile content of HMH. Globally, despite the few differences noted and specified previously, most of the compounds identified in HMH have already been reported in several marine products of different origins: fishing and aquaculture products [33,36,38,42,43], fish oil [21], fish sauces and fish pastes [25,34,39,44,45,46,47], and mollusk hydrolysates [9,48]. 

#### 3.1.2. Most Potent Odor-Active Volatile Compounds

To better understand the contribution of each compound to the overall aroma of the studied HMH, the frequency detection method was used. As shown in Table 3, a total of 15 compounds were perceived by at least 5 of the 13 judges, and would be considered to be the most potent odorants of the HMH. 11 of these 15 odorants were aldehyde compounds. In a general way, aldehydes impact significantly the overall aroma, due to their lower odor thresholds compared to other chemical groups. This means that only a small concentration of these compounds is necessary to make them perceptible [33]. Among the aldehyde compounds, 3-methylbutanal was perceived by all the panelists, while 2-methylbutanal was perceived by 6 of the 13 judges. 3-methylbutanal and 2-methylbutanal are generally generated from the Strecker reaction of leucine and isoleucine, respectively [21,45]. Both of these saturated aldehydes are characterized by burnt, malted, dark chocolate smells. 3-methylbutanal was identified as being the major aldehyde contributing to the odor of white herring [49]. Pentanal, hexanal, heptanal and octanal were sniffed by at least 6 of the 13 judges. These compounds are derived from lipid oxidation. On the one hand, pentanal and hexanal are respectively responsible for pungent and green smells, while green, rancid and fishy notes are ascribed to heptanal [21,34,36,38]. On the other hand, octanal is described as having citrus and orange peel smells, but also as having fatty and fishy smells [34,36,38]. The smell of octanal may probably depend on its concentration and its possible interactions with other compounds present in the matrix of interest. An interesting point was noticed by Liu et al. (2017) concerning hexanal. They noted that hexanal boosted the global fishy odor when it acted synergistically with other compounds, while its odor could not really be considered fishy when it stood alone [50]. Also, it is noteworthy that hexanal was already found in herring paste and in white herring [39,49]. (Z)-4-Heptenal was perceived by 7 of the 13 judges. This alkenal would be formed during lipid oxidation, and it is characterized by a fishy off-flavor [11,38]. In a general way, alkanal and alkenals were known to contribute to the fatty-oily, slightly rancid odor of marine products [36]. Methional was sniffed by 11 of the 13 judges, while benzaldehyde was sniffed by all the panel members. On the one hand, methional is an aldehyde sulfur-containing compound characterized by cooked potato notes, whose formation is associated to the Strecker degradation of methionine. Interestingly, it was the only sulfur-containing compound perceived by the panel. Surprisingly, other sulfur-compounds, such as methanethiol, dimethyl disulfide and dimethyl trisulfide, well-known for the considerable role they play in the odor of fishery products, were not identified in this study, while the two other sulfur-compounds, 2-acetylthiazole and benzothiazole, present in HMH were not sniffed by the panel [49,51]. Methional was already identified as an odorant compound in white herring [49]. On the other hand, benzaldehyde is an aromatic aldehyde characterized by almond, nutty or fruity aromas [38,46]. The two alkadienals, (E,E)-2,4-heptadienal and (E,Z)-2,6-nonadienal, were perceived by, respectively, five and 12 of the 13 judges. They both originate from lipid oxidation [35]. Aidos et al. (2002) found that (E,E)-2,4-heptadienal was representative of the oxidative status of herring oil [52]. (E,E)-2,4-heptadienal is characterized by fatty and fishy odors, while (E,Z)-2,6-nonadienal is reported to have a cucumber-like smell [21,34,38]. Nevertheless, it was reported that (E,Z)-2,6-nonadienal promotes a fishy off-flavor while it stands with other compounds [52]. Another group of chemical compounds that was well perceived by the panel was ketone compounds. More precisely, 2,3-pentadione, (Z)-6-octen-2-one and 2-nonanone were sniffed by at least nine of the 13 judges. The presence of ketones can, similarly to aldehyde compounds, be attributed to lipid and/or amino acid degradation. Iglesias et al. (2009) mentioned that 2,3-pentanedione could be even used as an indicator of lipid oxidation in chilled fish muscle [51]. 2,3-Pentadione is responsible for butter and fruity notes, while 2-nonanone is characterized by grass and green notes [9,34,49]. However, no data was found in the literature concerning the odor of (Z)-6-octen-2-one, and it seemed that this compound was never identified in marine products. Three hypotheses could be made to explain the perception of (Z)-6-octen-2-one in this study. The first one is that, as the hydrolysate studied in that case is an HMH, this compound may be a specific compound of this type of matrix. Nevertheless, as no study has been carried out so far regarding the volatile compounds of fish milt, to the best of our knowledge, it was not possible to confirm this first hypothesis. A second possible explanation could be that a wrong identification occurred. In fact, there is another ketone with a similar structure, 1-octen-3-one, that is highly reported in the literature for its fishy smell, but it was not identified in the case of this study [11]. Finally, the last explanation could be that the judges continued to perceive the odor of benzaldehyde, which was eluted just before (Z)-6-octen-2-one. Indeed, some studies already highlighted that the use of GC-O was not an exact science, and some mistakes could happen due to the fact that the odor detector of this method is the human nose [38,53]. Since (Z)-6-octen-2-one was identified by GC-MS with a match degree of 92%, and since no information was available in the literature regarding the duration of the smell of benzaldehyde, the hypothesis that seemed to be the most plausible was that (Z)-6-octen-2-one was a specific odor-active compound of HMH. It is of interest to note that (E,E)-3,5-octadien-2-one, a ketone compound identified in this study but not perceived by the panel, is well recognized to contribute to fatty-fishy odors in fishery materials [39]. Finally, 1-Methyl-1H-tetrazole was also a compound detected by at least five of the 13 judges. 1-Methyl-1H-tetrazole is a cyclic nitrogen-containing compound. Similarly to (Z)-6-octen-2-one, no information was found in the literature concerning the odor and the presence of this compound in seafood materials. Moreover, the judge responses did not allow us to clearly distinguish if the smell of 1-methyl-1H-tetrazole was negative or positive. Therefore, it could be supposed that 1-methyl-1H-tetrazole was a specific odor-active compound of HMH. Except for (Z)-6-octen-2-one and 1-Methyl-1H-tetrazole, all the compounds perceived by the panel were already reported for their contribution to the odor of various fishery materials [21,25,34,36,38,44,45,47]. Interestingly, no compound belonging to the alcohol group was detected by the judges, particularly the alcohol compound 1-penten-3-ol. In fact, this compound, well-known to contribute to the fishy smell, was identified in HMH. However, it was not perceived by the judges as a contributor to the overall aroma of this hydrolysate. This could be explained by the fact that alcohols generally do not have a huge contribution to the overall aroma of food products due to their high odor detection thresholds [21,33,36,47]. This also explained the fact that no alkane and alkene compounds were perceived by the panel members.

### 3.2. Deodorization by ED

#### 3.2.1. ED Parameters

##### pH

**Figure 2:**
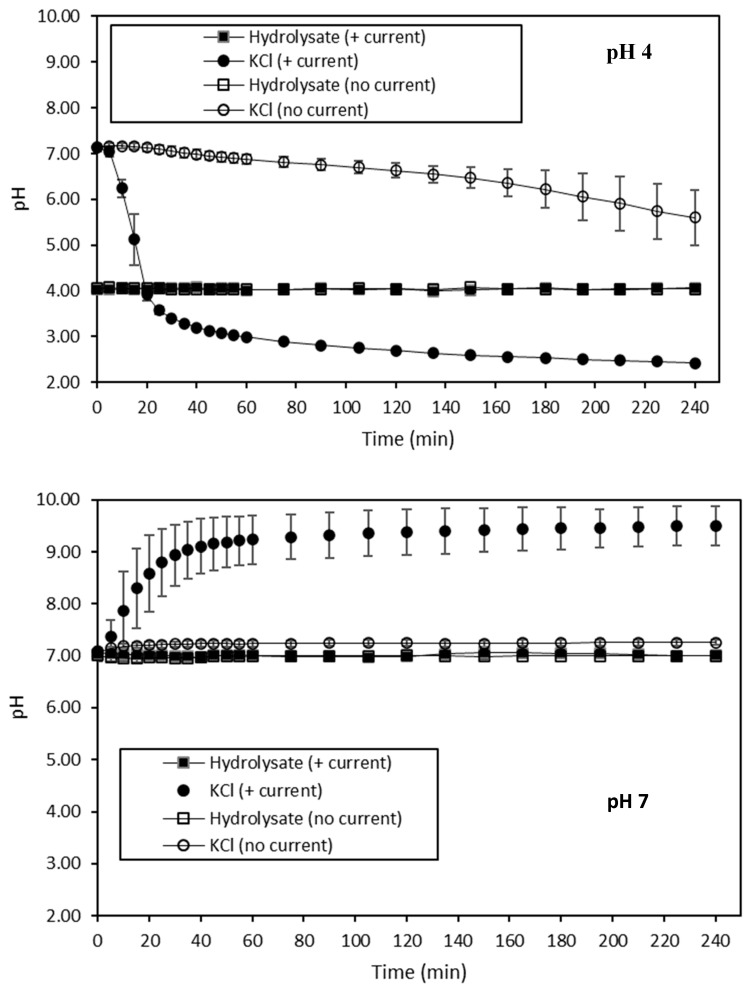
pH evolution in HMH solutions at pH 4 and 7, and in the corresponding KCl recovery solutions treated with and without current during ED treatments of 240 min.

The evolution of the pH of both HMH and KCl recovery solutions during ED treatments in the four different conditions is shown in Figure 2. Firstly, independently of the conditions of the current and pH tested, the pH of hydrolysate solution during the four different ED treatment conditions was steady at pH 4 or 7. However, concerning the KCl recovery solution, its pH varied differently according to the current conditions applied (*p* < 0.05), the pH of the hydrolysate (*p* < 0.05), and a combination of current conditions and pH (*p* < 0.05). Hence, for the hydrolysate at pH 4 without current, the pH of the KCl recovery decreased from 7.13 ± 0.06 to 5.60 ± 0.60 (*p* < 0.05), while those with current rapidly decreased from 7.13 ± 0.08 to 3.57 ± 0.08 (*p* < 0.05) during the first twenty minutes, and then continued to steadily drop to reach the final value of 2.42 ± 0.03. In the same time, regarding the treatment of hydrolysate at pH 7 without current, the pH of the KCl recovery solution remained steady at 7.06 ± 0.03 during all of the experiment, while with current, the pH of the KCl recovery increased rapidly from 7.09 ± 0.07 to 8.80 ± 0.65 (*p* < 0.05) during the first twenty minutes, and then continued to steadily rise to reach the plateau value of 9.50 ± 0.38.

The steady value of the pH observed for the hydrolysate solution during the four ED treatments was consistent with the fact that the pH of this solution was constantly adjusted to the desired value. The slight decrease in pH noticed for the KCl recovery solution of the hydrolysate at pH 4 without current would suggest the potential diffusion of acid species through the CEMs. Indeed, as HCl was added to acidify the hydrolysate at pH 4, H^+^ coming from the dissociation of HCl molecules diffused to the KCl solution. However, the rapid decrease in pH during the first twenty minutes of the experiment of the KCl recovery solution with current at the same pH could indicate that water dissociation took place at an early stage. Indeed, even if HCl molecules were initially added to lower and then to maintain the pH of the hydrolysate at pH 4, the fact that the pH of the corresponding KCl solution rapidly dropped below that of the hydrolysate implied that the electromigration of H^+^ acid species coming from the dissociation of HCl molecules would not have been sufficient to obtain such a decrease. For this reason, the occurrence of water dissociation could be considered. Water dissociation leads to the formation of protons and hydroxyl ions at the IEM (AEM or CEM) diluate interfaces, bringing about a pH variation [56,57]. This phenomenon generally occurs when the electrolyte concentration near the diluate side of the membranes becomes close to zero, and is due to the reaching of the limiting current density (LCD). As a consequence, the usual mass transfer of ionic species is hampered, and water dissociation takes place [27]. In that case, the decrease in pH value observed in the KCl recovery solution could suggest that the dissociation of water was more important at the CEM interfaces than at the AEM ones [56]. Regarding the KCl solution for the hydrolysate at pH 7 without current, where no variation of pH was observed, on the contrary to pH 4, no diffusion of H^+^ occurred. However, in that case, the quantity of HCl required to maintain the pH value of the hydrolysate at pH 7 was lower than that which was necessary to maintain the pH value at 4. This might explain the difference observed between pH 4 and 7 conditions. Finally, regarding the KCl recovery solution of hydrolysate at pH 7 with current, the drastic pH increase during the first twenty minutes was due to a rapid migration of basic species. Similarly to the ED experiment conducted on hydrolysate at pH 4 with current, water dissociation took place [32,58]. Nonetheless, in the present case, the increase in pH in the KCl recovery solution was related to the migration of OH^-^ basic species. This implied that the dissociation of water was more intensive at the AEM interfaces than at the CEM ones [32,56,58].

##### Conductivity

**Figure 3:**
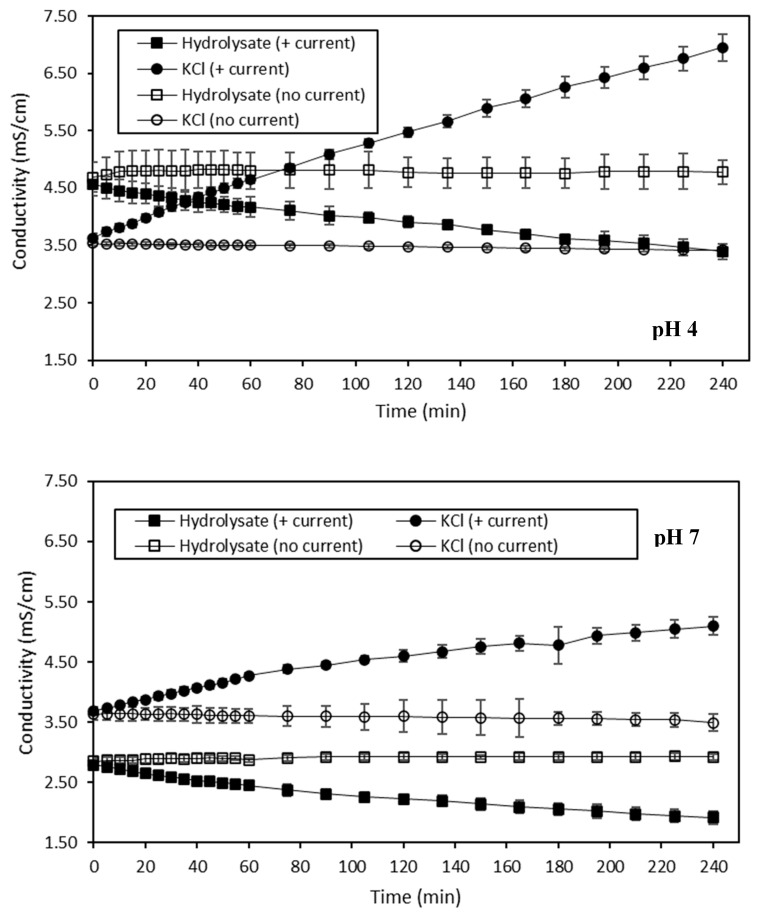
Conductivity evolution in HMH solutions at pH 4 and 7, and in the corresponding KCl recovery solutions, treated with and without current during ED treatments of 240 min.

The evolution of the conductivity of both HMH and KCl recovery solutions during the ED treatments in the four different conditions is presented in Figure 3. Firstly, the conductivity of the hydrolysate solution was mainly impacted by the current conditions (*p* < 0.05). The same trend was observed for the KCl recovery solution. Hence, when a current was applied on hydrolysate at pH 4, a decrease in the hydrolysate conductivity, from 4.57 ± 0.20 mS/cm to 3.40 ± 0.13 mS/cm, and an increase in the KCl solution conductivity, from 3.63 ± 0.07 mS/cm to 6.95 ± 0.13 mS/cm, were observed (*p* < 0.05). However, when the hydrolysate at pH 4 was treated without current, no change in conductivity of both the hydrolysate and the KCl recovery solutions was observed (*p* > 0.05). Regarding the ED treatment with current on hydrolysate at pH 7, a decrease in the conductivity of the hydrolysate, from 2.79 ± 0.06 mS/cm to 1.91 ± 0.11 mS/cm, and an increase in the conductivity of the KCl solution, from 3.69 ± 0.06 mS/cm to 5.10 ± 0.15 mS/cm, were also observed (*p* < 0.05), as for pH 4, but in a lower way. Finally, without current on hydrolysate at pH 7, no change was observed in the conductivity of both the hydrolysate and KCl recovery solutions, as for pH 4 (*p* > 0.05).

The decrease in conductivity observed for hydrolysate at pH 4 and 7 during the ED treatments conducted with current was representative of their demineralization, resulting in final respective DRs of 25.62 ± 0.92% and 31.45 ± 2.49%. Compared to other demineralizations performed by ED using a similar configuration, these two DRs were quite low. Indeed, in their studies, Dufton et al. (2018) and Lemay et al. (2019) reached a final DR of almost 70% for acid and sweet whey, respectively [58,59]. However, as it was already mentioned, the fact that, in this present study, the pH of the hydrolysate was constantly adjusted to the desired pH value with HCl hindered the demineralization process. In addition, in accordance with the pH evolution observed, some water dissociation took place rapidly after the first twenty minutes of both of these treatments, thus counteracting the efficiency of the demineralization. Nevertheless, the conductivity of hydrolysate at pH 4 was surprisingly quite high, to totally justify the possible occurrence of water dissociation suggested by the pH evolution. Indeed, Dufton et al. (2018) noted the occurrence of water dissociation at an acid whey conductivity close to 3.0 mS/cm [58], whereas, in the case of HMH at pH 4, the water dissociation would have begun at a conductivity close to 4.0 mS/cm. On the contrary, the fact that the conductivity of the hydrolysate at pH 7 was lower than 2.5 mS/cm made the occurrence of water dissociation due to the limited availability of ionic species for electric current transport even more plausible [27,60]. Regarding the two KCl solutions of the ED treatments performed with current, the increase in their conductivity values was correlated to the demineralization of the corresponding hydrolysate solutions, and thus to their mineralization, resulting in final MRs of 48.67 ± 0.50% and 27.65 ± 1.74% for the KCl recovery solution corresponding to the treatment of hydrolysate at pH 4 and 7, respectively. As a comparison, Dufton et al. (2018) obtained an MR of 74% for the recovery compartment of their study dealing with the demineralization of acid whey [58]. The lower MRs obtained in the case of this study were in line with the lower DRs of the hydrolysate discussed previously. Moreover, the conductivity of the KCl solution for hydrolysate at pH 4 with current increased linearly. This suggests the continuous migration of ionic species, among them H^+^ species, into the KCl compartment. However, it was less obvious to qualify the evolution of KCl conductivity as linear for the ED treatment conducted on hydrolysate at pH 7 with current, indicating that the H^+^ migration would not be as continuous in that case. Finally, the absence of changes in the conductivity of the hydrolysate solutions at pH 4 and 7, and the corresponding KCl recovery solutions for the ED treatments conducted without current, showed that no demineralization and no mineralization occurred. This was in line with the fact that no current was applied. Concerning ED on the hydrolysate at pH 4 without current, the diffusion of H^+^ suggested by the pH evolution was not perceived in terms of conductivity in that case. This observation was not consistent with the fact that H^+^ species are known to impact conductivity [61]. However, the observed drop of pH was approximately of one unit, meaning that this variation was caused by around 0.000001 M of H^+^ and, normally, the conductivity contribution of this ionic species would not have been perceived in 0.02678 M of 2 g/L KCl. The fact that such pH variation was visible suggests that the membrane integrity could have been altered by a potential fouling.

##### Ash Content

The ash content of HMH and KCl recovery solutions was analyzed, and is presented in Table 4 and Table 5. The ash content of the hydrolysate solution was only impacted by the current conditions applied (*p* < 0.05). The same trend was observed for the KCl recovery solution. At the initial time, the ash content of hydrolysate at pH 4 was 0.478 ± 0.005%. Hence, after ED with current, the ash content of the hydrolysate at pH 4 decreased to 0.400 ± 0.048% (*p* < 0.05), while no change was observed regarding the ash content of hydrolysate at pH 4 after ED without current (*p* > 0.05). Concerning the hydrolysate at pH 7, its ash content was 0.484 ± 0.007% at the initial time. This value was similar to those obtained for hydrolysate at pH 4 at the initial time (*p* > 0.05). After ED of the hydrolysate at pH 7 with current, the ash content decreased to 0.427 ± 0.020% (*p* < 0.05). The final ash contents of both hydrolysates at pH 4 and 7 after ED with current applied were similar (*p* > 0.05). However, ED carried out without current did not lead to any change in the ash content of the hydrolysate at pH 7 at the final time compared to the initial time (*p* > 0.05). With regard to the KCl recovery solution, its initial ash content was 0.166 ± 0.007%. After ED with current on the hydrolysate at pH 4, the ash content of the KCl recovery solution rose to 0.240 ± 0.022% (*p* < 0.05). At the same time, the ash content of the KCl recovery solution for ED with current on hydrolysate at pH 7 increased as well, compared to the initial time, since the final value obtained in that case was 0.230 ± 0.005% (*p* < 0.05). The ash content of both KCl recovery solutions after ED with current on the hydrolysate at pH 4 and pH 7 was similar (*p* > 0.05). Finally, no change was observed in the ash content of KCl recovery solutions after ED of the hydrolysate at pH 4 and 7 without current, compared to initial time (*p* > 0.05).

The ash content of the hydrolysate at pH 4 and the hydrolysate at pH 7 at the initial time was surprisingly similar. Indeed, as the acidification of the hydrolysate to pH 4 required more HCl than the acidification to pH 7, it would have been more logical to obtain a higher value of ash content for the hydrolysate at pH 4 than for the hydrolysate at pH 7, due to the contribution of Cl^-^ species formed by the dissociation of HCl molecules. Nevertheless, the fact that the difference in HCl volume added to the initial hydrolysate to reach these two pH values was only in the order of a few milliliters might explain this result. The lower ash content of the hydrolysate at pH 4 and 7 after ED with current was related to their demineralization. This observation was in line with the decrease in the conductivity of these two solutions, as described previously. Based on the initial and final ash content values, the DRs obtained for the hydrolysate at pH 4 and for the hydrolysate at pH 7 was 16.71 ± 4.59% and 11.62 ± 4.71%, respectively. However, these two DR values were not consistent with those of 25.62 ± 0.92% for the hydrolysate at pH 4 and those of 31.45 ± 2.49% for the hydrolysate at pH 7, determined by means of the conductivity measurements. This discrepancy between the DR values would confirm the occurrence of water dissociation during these two ED treatments. Indeed, it was already observed that the calculation of DR based on conductivity values could be biased in the case of water dissociation, since this phenomenon leads to the formation of ionic species, namely H^+^ and OH^-^, impacting the conductivity but not the ash content [61]. Regarding the corresponding KCl solutions, their increase in ash content was correlated to their mineralization. This was consistent with the increase in their conductivity mentioned before. For the KCl recovery solutions, based on their ash content values, the corresponding MRs were 29.27 ± 3.40% and 28.87 ± 3.24% for the hydrolysate at pH 4 and 7, respectively. However, the MRs obtained in the case of the conductivity measurements were 48.67 ± 0.50% and 27.65 ± 1.74% for the KCl recovery solutions after ED of the hydrolysate at pH 4 and 7, respectively. If the MRs of the KCl recovery solution of the hydrolysate at pH 7 were similar in both cases, at pH 4 they were highly different depending on the equation used to calculate them. This could suggest that a higher migration of H^+^ species took place in the KCl recovery solution during ED of the hydrolysate at pH 4 with current. As explained previously, the MR of the KCl recovery solution was overestimated, based on conductivity values [61]. This could also explain why the conductivity evolved linearly for the KCl solution during ED with current of the hydrolysate at pH 4, but not for the KCl with current at pH 7. Finally, the fact that the ash content of the hydrolysate at pH 4 and 7 after the ED treatments without current remained unchanged compared to initial time was in line with the absence of demineralization, since no current was applied. This also explained the fact that no change was observed in the ash content of the corresponding KCl solutions.

##### Global System Resistance

**Figure 4:**
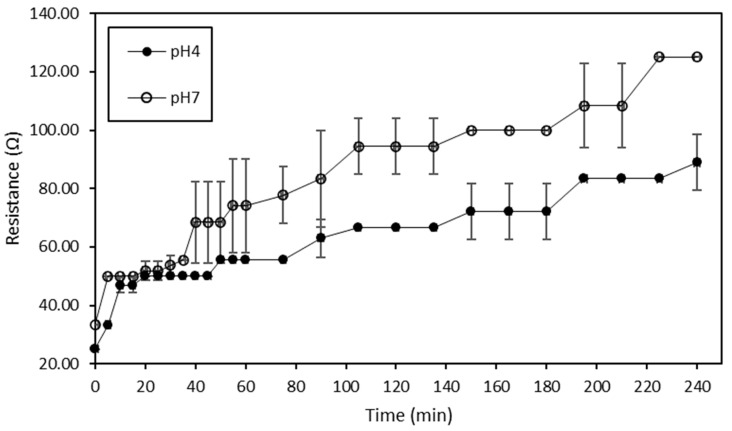
Global system resistance evolution during ED treatments of 240 min at pH 4 and pH 7, with current.

The global system resistance evolutions of ED treatments conducted at pH 4 and 7 with current are shown in Figure 4. Regarding the treatment conducted at pH 4, the global system resistance increased significantly from 25.00 ± 0.00 Ω to 88.89 ± 9.62 Ω (*p* < 0.05), while those concerning the treatment carried out at pH 7 increased significantly from 33.33 ± 0.00 Ω to 125.00 ± 0.00 Ω (*p* < 0.05). Moreover, for both treatments, the increase in global system resistance was even more visible after the first twenty minutes of treatments.

The ED treatments performed on the hydrolysate at pH 4 and 7 presented a 3.5-fold increase and a 3.75-fold increase in global system resistance, respectively. Dufton et al. (2018) experienced a similar increase in global system resistance during the demineralization of acid whey by ED [58]. Such an increase in global system resistance could not only be due to the demineralization process but also to the occurrence of water dissociation as a cause or as a consequence of potential membrane mineral and/or protein fouling, as previously observed by Dufton et al. (2018) [58]. This is corroborated by the fact that this increase in global system resistance for both ED treatments took place at the same time as the changes in pH evolution mentioned previously. Another interesting point to mention was that, during ED performed on hydrolysate at pH 7, the intensity dropped considerably after the first twenty minutes (data not shown). The decrease in intensity was representative of a lack of ionic species to carry the electric current, and was thus consistent with the increase in global system resistance that was even more visible after the first twenty minutes of treatment. This observation was in line as well with the lower conductivity of the hydrolysate at pH 7 noted rapidly after the beginning of the treatment, suggesting that the LCD was reached. This could explain why the global system resistance was, in that case, so high. However, the fact that the global system resistance of ED conducted on the hydrolysate at pH 4 was lower could indicate that, at that stage, it was still not possible to clearly identify whether the LCD was reached and thus explain the water dissociation.

##### Membrane Thickness

**Figure 5:**
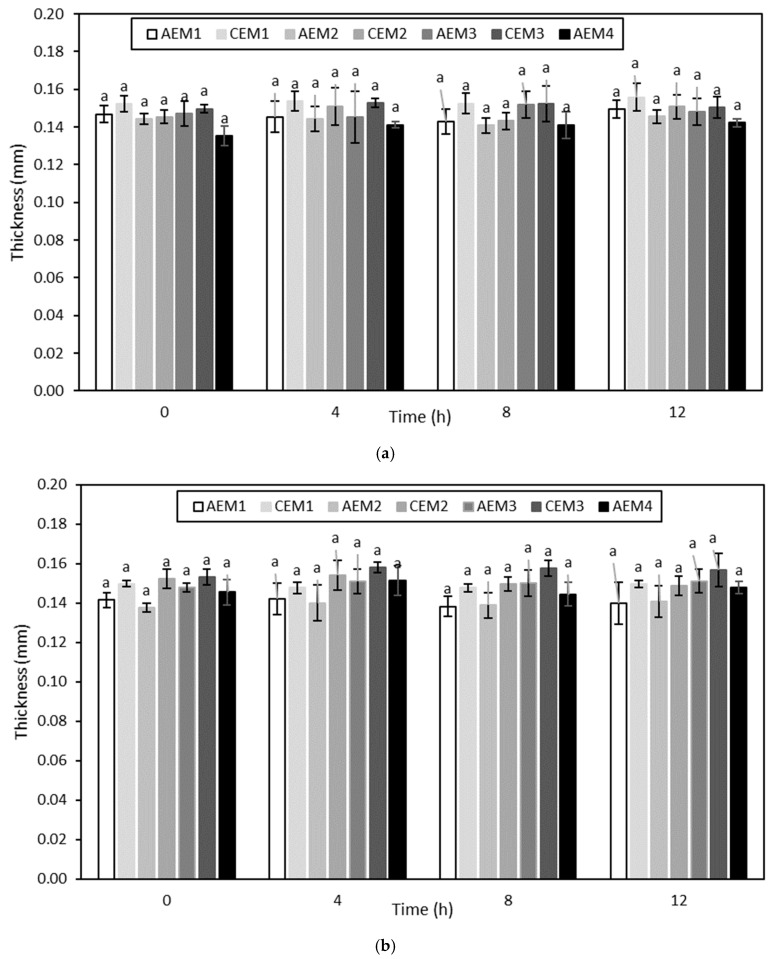
Membrane thickness before and after each 4-h ED treatment conducted at pH 4, (**a**) without current and (**b**) with current. Values with different letters corresponding to the same membranes are significantly different *p* < 0.05 (Tukey test).

**Figure 6:**
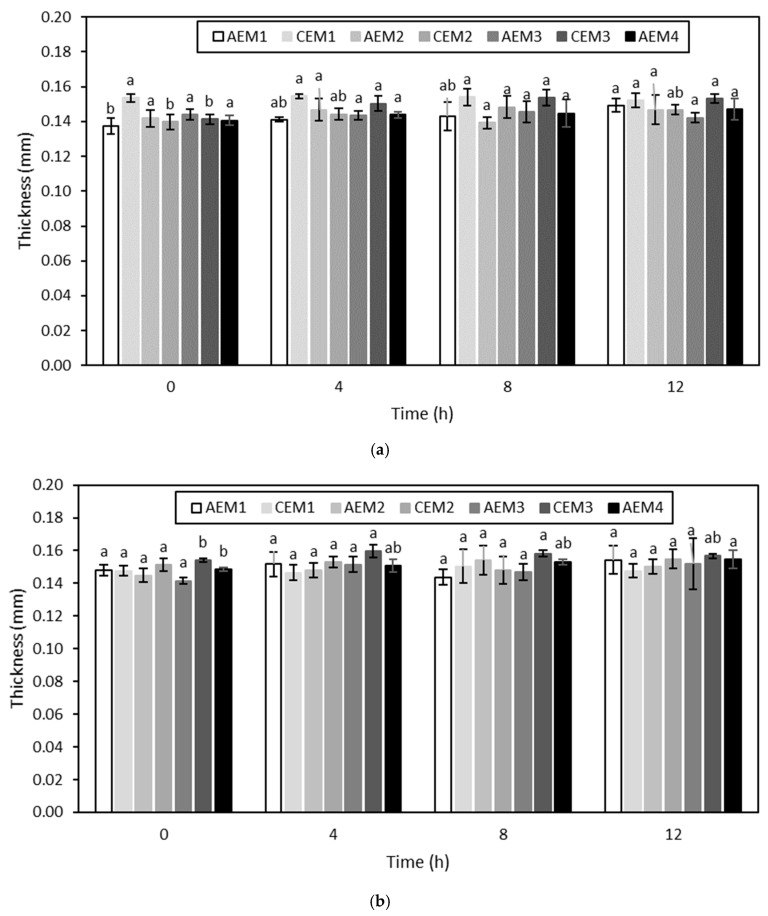
Membrane thickness before and after each 4-h ED treatment conducted at pH 7, (**a**) without current and (**b**) with current. Values with different letters corresponding to the same membranes are significantly different, *p* < 0.05 (Tukey test).

The evolution of the membrane thickness over the different ED treatments is shown in Figure 5 and Figure 6. None of the membranes evidenced an increase in thickness over ED, with and without current, performed on the hydrolysate at pH 4 (*p* > 0.05) (Figure 5). Regarding the ED treatments carried out on the hydrolysate at pH 7, both AEM1 and CEM3 showed an increase in thickness (*p* < 0.05) for the experiment without current, while only AEM4 presented an increase in thickness (*p* < 0.05) for the experiment with current (Figure 6).

Membrane thickness is an indicator of membrane integrity, and more particularly of membrane fouling [58]. This could indicate that no fouling occurred during ED treatments on the hydrolysate at pH 4, showing the efficiency of the NaCl rinsing after each run. This was consistent with the visual observations of the membranes after each run as well. However, even if a few membranes evidenced an increase in thickness after the ED treatments on the hydrolysate at pH 7, the final membrane thickness values were still representative of those reported in the literature. Indeed, Lemay et al. (2019) reported average values of 0.142 ± 0.006 mm and 0.141 ± 0.005 mm for AEMs and CEMs, respectively [32]. Therefore, based on membrane thickness evolution, no fouling phenomena seemed to happen at that stage regarding the treatments on the hydrolysate at pH 7.

##### Membrane Conductivity

**Figure 7:**
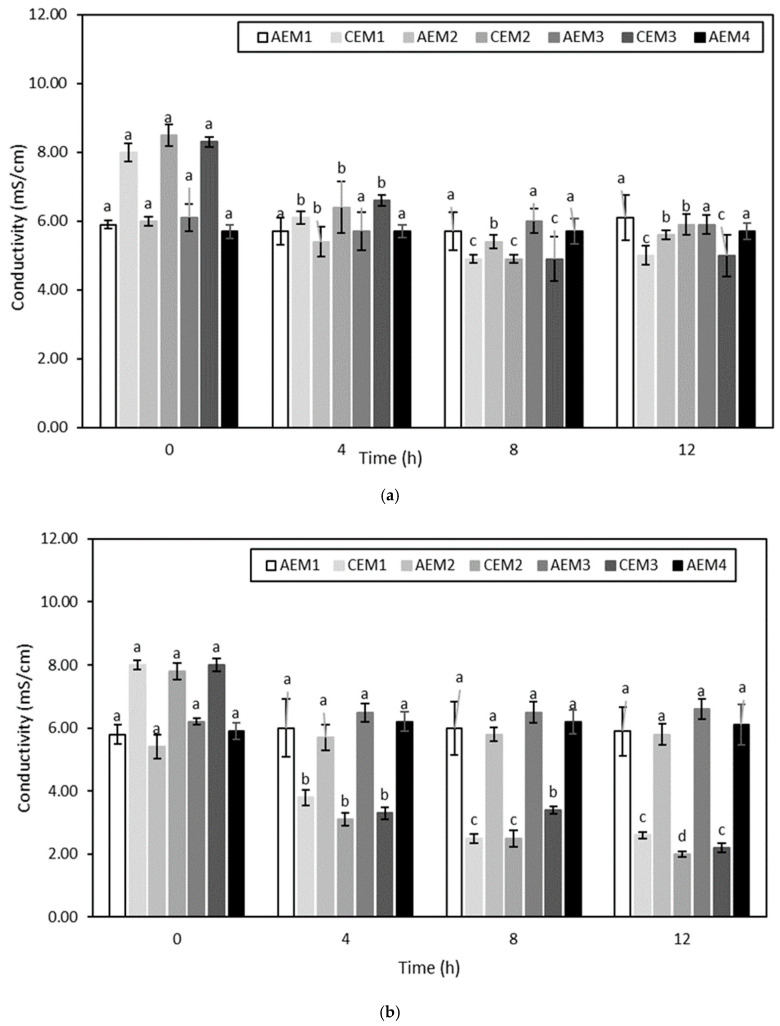
Membrane conductivity before and after each 4-h ED treatments conducted at pH 4, (**a**) without current and (**b**) with current. Values with different letters corresponding to the same membranes are significantly different *p* < 0.05 (Tukey test).

**Figure 8:**
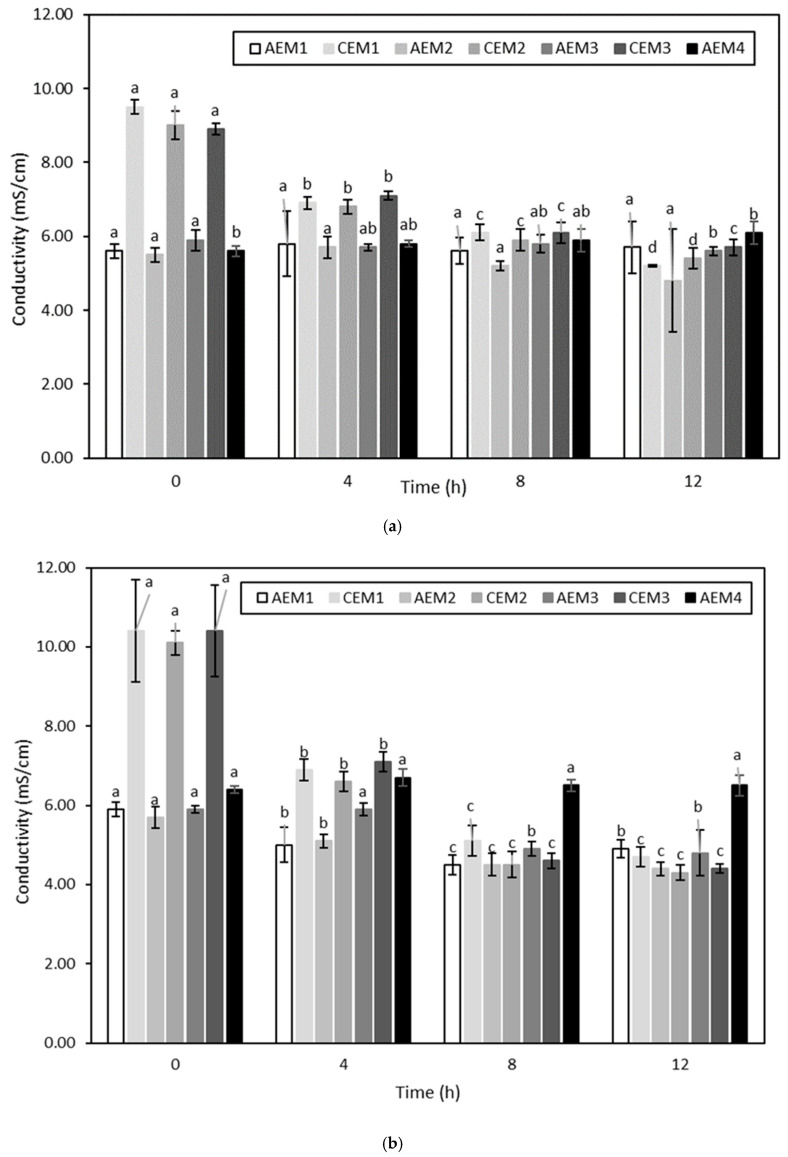
Membrane conductivity before and after each 4-h ED treatments conducted at pH 7, (**a**) without current and (**b**) with current. Values with different letters corresponding to the same membranes are significantly different *p* < 0.05 (Tukey test).

The membrane conductivity evolution over the different ED treatments is presented in Figure 7 and Figure 8. Regarding ED conducted on the hydrolysate at pH 4, AEM2 and all the CEMs for the experiment conducted without current, and all the CEMs only for the experiment conducted with current, evidenced a decrease in their conductivity over time (*p* < 0.05) (Figure 7). Concerning ED on the hydrolysate at pH 7, AEM3, AEM4 and all the CEMs for the experiment without current, and all the AEMs except AEM4 and all the CEMs for the experiment using current, experienced a decrease in their conductivity over time (*p* < 0.05) (Figure 8).

Similarly to membrane thickness evolution, the evolution of membrane conductivity can be considered to be an indicator of membrane integrity [58]. Firstly, the conductivity of both CEMs and AEMs before any run was comparable to the following values reported in the literature: 5.197 ± 0.257 ms/cm and 8.960 ± 0.442 mS/cm for AEMs and CEMs, respectively [32]. Then, all the four ED conditions evidenced a change in membrane conductivity as a function of time. In their study, Lemay et al. (2019) also noted a decrease in membrane conductivity after sweet whey demineralization [32]. In that case, the observed drop was attributed to the substitution of the counterions present in the initial membranes by divalent ionic species of the sweet whey having lower conductivity values, resulting in a decrease in membrane conductivity [32]. Nonetheless, in the present study, the fact that membranes evidenced a decrease in conductivity not only after the ED treatments conducted with current but also after those performed without current may suggest that another explanation could be involved. More specifically, regarding the ED treatments carried out on hydrolysate at pH 4, a decrease in membrane conductivity was already noted after the first run. This could indicate that, independently of the current conditions, ionic compounds present in the hydrolysate solution interacted with the membranes. Indeed, previous works have already showed that charged compounds such as peptides and amino acids could interact with the boundary layers of membranes [56]. As the decrease in membrane conductivity was mainly observed for CEMs, this means that the compounds involved were cationic and interacted with the negatively charged sulfonic groups present in the CEMs. More specifically, Persico et al. (2017) showed in their study the ability of peptides containing histidine (pKa of 6.0), lysine (pKa ~10.5) and arginine (pKa ~12.5) residues, in addition to their amine group at the N-term (pKa ~9.8), to interact electrostatically with the negatively charged sulfonic groups of CEMs due to the positive charges they carry, even when no current was applied [62]. Free arginine could even be considered to be a major agent responsible for the fouling of CEMs [63]. Since HMH is mainly composed of amino acids containing materials (Table 1) including a high amount of arginine in both bound and free forms [1], the important decrease observed in the CEM conductivity could be explained by the neutralization of the fixed membrane charges by these molecules, among them arginine amino acid. At that stage, it is worth it to mention that, after each run, NaCl rinsing was performed. The aim of such rinsing was to reduce the electrostatic interactions occurring between the ionogenic constituents of the membranes and amino acids containing materials from the hydrolysate due to the high ionic strength of the salt rinsing solution [64]. However, the fact that fouling was observed even after this rinsing indicates that other interactions than electrostatic ones could be involved between the ionogenic groups of membranes and components from the hydrolysate. This suggests that hydrophobic interactions could be involved between volatile compounds and membrane constituents as well, as that type of interaction was not impacted by NaCl rinsing [64]. This hypothesis was already formulated in the studies of Cros et al. (2005) and Chindapan et al. (2011), in which they ascribed the decrease in the abundance of certain volatile compounds to hydrophobic interactions occurring with membrane components [25,26]. In addition, the observed decrease in membrane conductivity could be explained by another phenomenon. Indeed, it was already shown that, depending on the nature of the groups from both the membranes and the matrix present at the membrane interface, the catalysis of water dissociation during an ED treatment could take place even if the LCD was not reached [56,65]. More precisely, at the CEM interface, the catalytic action of carboxylic acid present at the C-term of the peptides was already proven [56]. Therefore, the decrease in pH observed in the KCl recovery solution corresponding to the ED treatment of the hydrolysate at pH 4 with current, and the increase in its conductivity, could be effectively due to the occurrence of water dissociation resulting from the action of cationic catalysts compounds, such as peptides or free amino acids involving a carboxylic group, at the boundary layers of the CEMs. On the contrary, for ED of the hydrolysate at pH 4 without current, since a decrease in the CEM conductivity was evidenced, but with no huge pH change of the KCl solution, this could suggest that the interaction of cationic compounds took place, resulting in membrane fouling without bringing about water dissociation due to the absence of current. Regarding ED on the hydrolysate at pH 7, since both AEMs and CEMs presented a decrease in conductivity for both current conditions applied, this may suggest not only that cationic compounds interacted with CEMs but also anionic ones with ammonium groups of AEMs. This observation was consistent with the study of Persico et al. (2016), in which it was observed that fouling of AEMs was absent at acidic pH, while it tended to be more important at a pH close to neutral [64]. This was due to the fact that, at a pH close to neutral, negative residues of carboxylic acid present at the C-term (pKa ~2.1) of peptides, or at the side-chains of aspartic and glutamic acids (pKa ~4.0), were able to interact electrostatically with the positively charged ammonium groups of AEMs [64]. As both aspartic and glutamic acids are present in HMH, in both bound and free forms [1], they could have been responsible for the fouling observed on the AEMs due to their carboxylic acid residues leading to the observed decrease in conductivity. Nevertheless, as noted for CEMs, the fact that fouling was evidenced even after the NaCl rinsing could suggest that other interactions than electrostatic ones could be involved. Moreover, similarly to what was mentioned previously as well, water dissociation phenomena could have taken place at the interface of AEMs, due to the catalytic action of both compounds present in the AEMs and in the hydrolysate [56,65,66]. More precisely, the conversion of the initial quaternary ammonium groups (-N^+^(CH_3_)_3_) present in the AEMs into tertiary (=N(CH_3_)_2_) or secondary amine (≡N(CH_3_)) is a factor accelerating water dissociation due to the lone electron pair carried by the newly formed groups [56,66]. Weak-acid anions [65], such as glutamic and aspartic residues present in the studied hydrolysate, can catalytically accelerate the water dissociation as well. Furthermore, this hydrolysate also contains nucleic acids that carry a negative charge due to the presence of phosphoric acid in its anion form. Therefore, nucleic acids could have also established interactions with AEMs, or could have played a role in the catalysis of water dissociation. At this stage, it is worth to mention that the occurrence of water dissociation due to the generation of H^+^ and OH^-^ could be a factor promoting membrane fouling by increasing the interaction with amino-containing materials at the membrane interface [67]. In addition, the fact that both types of IEM were fouled for the condition at pH 7 could explain why the global system resistance increased more significantly than those during the ED treatment conducted on the hydrolysate at pH 4. Another interesting point to note is that the standard deviations corresponding to the global system resistance of the ED treatment of the hydrolysate at pH 7 were quite important compared to those displayed for the ED treatment of the hydrolysate at pH 4. This would probably be due to the progressive decrease in conductivity observed for both types of IEM. It is worth to mention that at that stage, the potential interactions occurring between constituents of the studied hydrolysate, including volatile compounds and the IEM, seemed to be the most plausible explanation. Therefore, the occurrence of water dissociation observed during the ED treatment of the hydrolysate at pH 4 with current could be due more to the action of catalysts than to the reaching of the LCD.

#### 3.2.2. Hydrolysate and Recovery Solutions Analyses

##### Volatile Compound Analysis

Most Potent Odor-Active Compounds

The abundance of compounds determined to be the most potent odorants of the HMH over the different ED and deaerator treatments is shown in Table 6. Firstly, concerning the composition of the hydrolysate at the initial time, the results indicate that pH had an important impact on the volatile compounds’ abundance. It appeared that the abundance of the majority of the most potent odor-active molecules significantly decreased while pH increased from 4 to 10. For example, the abundance of 3-methylbutanal, 2,3-pentanedione, pentanal, hexanal, (Z)-4-heptenal, heptanal, methional, (Z)-6-octen-2-one, (E,E)-2,4-heptadienal, octanal, 2-nonanone and (E,Z)-2,6-nonadienal dropped by at least 50% between the hydrolysate at pH 4 and the hydrolysate at pH 10 at the initial time (*p* < 0.05). 1-methyl-1H-tetrazole was the only compound that did not follow this trend. In fact, it was identified in the hydrolysate at pH 7, but it was not present in the hydrolysate at pH 4 and pH 10. In general, pH is known to be a major factor influencing the content of volatile compounds. Indeed, volatile compounds are able to interact with molecules like lipids through hydrophobic interactions, and amino acids constituents such as proteins, peptides and free amino acids through covalent irreversible bindings, in addition to hydrophobic and ionic interactions [68,69,70]. Among these different interactions, those taking place between volatile compounds and amino-acid containing compounds are the most impacted by pH, as this factor modifies the conformation and charge of proteins, peptides and free amino acids, and thus the ability of binding of volatile compounds [69]. Based on this fact, two hypotheses can be made concerning the general decrease in volatile compounds observed while pH increased. The first could be that alkaline pH might be responsible for the breaking of interactions taking place between volatile compounds and amino groups. As the targeted compounds are volatile, breaking these interactions could promote their loss. On the contrary, the second could be that the lower abundance of volatile compounds observed in the HMH at pH 10 at the initial time may be representative of a higher interaction with amino acid materials. As no study has been carried out regarding the impact of pH on the retention of volatile compounds by amino acid constituents from HMH materials so far, it was not obvious to clearly validate one hypothesis rather than the other. Nevertheless, some studies with similar purposes were already conducted on milk proteins [71,72], as well as on animal tissues proteins and peptides [69,73]. These studies showed that there was a general trend of amino acid containing molecules, such as peptides, to retain volatile compounds to a higher extent while pH increased. Several explanations are involved, depending on the proteins. For example, the milk protein β-lactoglobulin is reported to bind a larger proportion of volatile compounds at pH 9 than at pH 3. The increase in retention ability is, in this case, explained by better access to the hydrophobic amino acid residues of β-lactoglobulin due to conformation changes occurring under alkaline conditions [70,72]. Interestingly, leucine, a hydrophobic amino acid, is present in both β-lactoglobulin and HMH. Leucine is even present in high proportion in the latter (Table 1). It could be suggested that leucine might participate in the retention of volatile compounds, and while pH increases, the loss of the proton H^+^ on the amine group could promote this phenomenon. Independently of pH, Meynier et al. (2004) observed the unavailability of lysine and histidine of milk proteins in the presence of aldehydes, suggesting a potential interaction occurring between these amino acids and volatile compounds. It was proposed to explain this loss that the carbonyl group of aldehydes could react with the primary amine of lysine either by Michael addition or by Schiff base formation. Concerning histidine, it was suggested that aldehyde and, preferentially, alkenal could react with the imidazole ring of histidine [74]. Since among the 15 compounds identified as being the most potent odor-active of HMH, 11 are aldehydes and one of them is an alkenal, namely (Z)-4-heptenal, and since lysine and histidine are both present in this product (Table 1), it could be possible that these interactions occur between these volatile compounds and these amino acids. Histidine is also a constituent of carnosine, a dipeptide found in animal tissues, and whose ability to retain volatile compounds is also known to increase while pH increases [69]. In that case, as pH affects the retention of volatile compounds, it could be proposed, similarly to leucine, that the loss of the proton H^+^ on the imidazole ring occurring under alkaline conditions could promote the interaction between histidine and aldehydes. Interestingly, HMH contains a high amount of arginine (Table 1), which is an amino acid with an amine-containing side-chain similar to lysine and histidine. Based on this, it should be proposed that arginine was also involved in the interactions, explaining partially the decrease in volatile compound abundance observed. Therefore, the hypothesis that seemed to be the most plausible regarding the decrease in the abundance of volatile compounds at pH 10 compared to pH 4 and pH 7 would be those implying a higher degree of interaction occurring between volatile compounds and amino acid containing compounds, such as peptides present in the hydrolysate. In that case, the fact that 1-methyl-1H-tetrazole was not detected in the hydrolysate at pH 4 and 10 might suggest that this compound could have more interactions at these pH values than at pH 7, allowing its detection at this pH value only. Also, it is of interest to mention that, while volatile compounds are bound to other components, both their release and perception are hindered [69]. This means that HMH should be globally less odorous at pH 10 than at pH 4 and pH 7.

Then, regarding the ED treatments, no significant difference was globally observed between the hydrolysate at a given pH at the initial time and the hydrolysate treated with or without current at the final time. More precisely, if attention is paid to the ED treatments conducted without current, the fact that no decrease in the content of the targeted volatile compounds was observed would suggest that a simple circulation of the hydrolysate solution for 240 min, independently of its pH, was not sufficient enough to allow a loss of these molecules due to their volatile state. Concerning ED conducted with current, no change in the content of volatile compounds occurred except for (Z)-6-octen-2-one, (E,E)-2,4-heptadienal and (E,Z)-2,6-nonadienal whose abundance was inferior at final time for ED on the hydrolysate at pH 4 (*p* < 0.05). These results could be representative of the non-migration of volatile compounds. Since ED is a process based on the migration of charged compounds, and since the targeted compounds were not supposed to be charged under the conditions tested, it was not surprising, at the first glance, to obtain such results. However, in their studies, Cros et al. (2005) and Chindapan et al. (2011) observed that an ED treatment could lead to a drop of volatile compounds even if they are not charged [25,26]. Several points could explain the discrepancy between these two studies and the present one. The first could be that the compounds whose abundance dropped during ED treatments in the studies of Cros et al. (2005) and Chindapan et al. (2011) were not the same compounds targeted in the present study. Indeed, Cros et al. (2005) observed a significant decrease in the non ionizable (Z)-4-heptenal, 2,3-butanedione, 3-octen-2-one and limonene compounds. The only compound that this study and the present one had in common was (Z)-4-heptenal. Nevertheless, it is noteworthy to mention that Cros et al. (2005) noticed the important decrease of the compounds listed before only while the LCD was reached. Different hypotheses were formulated to explain such a decrease under this specific condition in this study. The first was that the formation of protons H^+^ and hydroxyls OH^-^ resulting from water dissociation under this critical condition could have altered volatile compounds, explaining their decrease. Another was that the LCD could have also brought about a local membrane heating, potentially leading to a thermal degradation of volatile compounds. Or, simply, the volatile compounds could have been adsorbed on the membranes through hydrophobic and ionic interactions [26]. The migration of these molecules was not considered to be a potential explanation, as none of them were found in the recovery solution. Regarding (Z)-4-heptenal only, Cros et al. (2005) hypothesized its hydrogenation in heptanal as a possible explanation for its decrease [26]. Since not all the ED treatments conducted in the present study seemed to have evidenced reaching LCD, it was not possible to totally verify all the hypotheses formulated by Cros et al. (2005). However, as suggested by the analyses of the parameters of the ED treatment conducted on the hydrolysate at pH 7, it seems that this condition experienced the reaching of LCD. As no change regarding the volatile compound content was observed in that case, it may indicate that the LCD was not a sufficient condition to lead to a decrease in the abundance of these compounds. In addition, the fact that both ED treatments conducted with current presented water dissociation in the present study could show that the generation of H^+^ and OH^-^ species could not be effectively responsible for the alteration of the volatile compounds. On the contrary to Cros et al. (2005), Chindapan et al. (2011) did not reach the LCD condition in their study. Despite this fact, they observed a significant decrease in 2,6-dimethylpyrazine, phenol and carboxylic acids (acetic acid, butanoic acid, 2-methylbutanoic acid, pentanoic acid, 4-methylpentanoic acid) while the ED treatment was performed, to reach a salt concentration of 2% in the treated fish sauce. Chindapan et al. (2011) gave two main reasons for the loss of these compounds: either their adsorption on the membranes or their transport through the membranes occurring at the same time as electroosmosis. Nevertheless, as no mention concerning the composition of the recovery solution was made, it was not possible to know if the latter reason was plausible in that case [25]. Concerning the decreased in (Z)-6-octen-2-one, (E,E)-2,4-heptadienal and (E,Z)-2,6-nonadienal observed in the hydrolysate at pH 4 treated with current at final time, three hypotheses could be made, based on those previously mentioned. The first one would be that a slight loss of these molecules due to their volatile state happened during the ED process. However, as no decrease of these compounds was observed for other conditions, this hypothesis does not seem highly plausible. The second hypothesis would be that preferential interactions occurred at pH 4 between these three volatile compounds and other constituents of the HMH. Finally, the last hypothesis would be that these compounds preferentially adsorb on the membranes due to hydrophobic interactions. This last hypothesis appears to be the most probable, based on the membrane conductivity evolution discussed previously.

The content in volatile compounds of the KCl recovery solutions was analyzed for each condition, and is listed in Table 7. The results show the unchanged presence of 3-methylbutanal, hexanal, heptanal, benzaldehyde, octanal and 2-nonanone in KCl solution at final time independently of the ED treatment. It is worthwhile to mention that none of these compounds were detected in the KCl solution at the initial time. Except for benzaldehyde and hexanal, in all cases, the presence of volatile compounds in the recovery solution can be considered to be trace. This should probably be due to a punctual contamination of these compounds due to their volatility from the hydrolysate to the KCl solution. The fact that this phenomenon could be considered to be punctual was accredited by the generally high values of standard deviations proportionally to those of means, and even sometimes the higher values of standard deviations compared to the corresponding means. However, another explanation could be involved for hexanal and benzaldehyde. Regarding hexanal, its presence in the KCl solution could be due to its diffusion or migration. However, based on the membrane conductivity analysis discussed previously, it seemed that some interactions with membrane components also occurred during the different ED treatments. Therefore, another explanation could be that hexanal may have interacted with the sulfonic groups present in the CEMs, resulting in its release into the KCl compartment thereafter. Interestingly, the same trend was not found for compounds similar to hexanal, such as pentanal and heptanal. In that case, the differences observed should probably be due to the presence of hexanal in higher quantity in HMH, compared to pentanal and heptanal. Regarding benzaldehyde, the same explanations as those mentioned for hexanal could be involved. Nevertheless, on the contrary to hexanal, the fact that benzaldehyde was found in higher abundance only in KCl solution of the hydrolysate at pH 4 treated with current may indicate that a special mechanism was involved in that case. Initially, as benzaldehyde is not charged, it was not supposed to migrate. However, its recovery in the KCl solution might suggest that benzaldehyde could have either established interactions with another positively charged constituent that migrated into the CEMs, or that benzaldehyde established an interaction with the sulfonic groups of the CEMs, resulting in its release into the KCl compartment thereafter. Nonetheless, assuming that an interaction with another constituent could explain the presence of benzaldehyde in that case, this interaction could have been broken once this compound was finally in the KCl solution, as its detection was still allowed. Indeed, as mentioned previously in this study, while volatile compounds interact with other constituents, it hinders their detection [69]. Moreover, the results could show that this potential interaction occurred only at pH 4, as a similar trend was not found at pH 7. The charged compounds in that case could be histidine, present mainly in its free form in the HMH [1] as, at pH 4, its side-chain was totally protonated (pKa ~6.0), allowing its migration to the cathode through CEMs, while at pH 7 this latter was in its non-charged form. Interestingly, the presence of (Z)-6-octen-2-one, (E,E)-2,4-heptadienal and (E,Z)-2,6-nonadienal, whose abundance was lower in the hydrolysate at pH 4 treated with current at the final time, was not found in the corresponding KCl solution. Therefore, supposing that the hypothesis formulated before aiming that these compounds could have established interactions with membranes, this could indicate that none of these three compounds were released into the KCl compartment thereafter.

Finally, the performance of ED to decrease the abundance of the most potent odor-active compounds of the HMH was compared to that of a deaerator (Table 4). In this case, in addition to pH 4 and 7, the treatment was also conducted on the hydrolysate at pH 10. Compared to pH 4-hydrolysate at the initial time, the deaerator allowed the decrease in seven compounds (*p* < 0.05), namely 3-methylbutanal, 2-methylbutanal, 2,3-pentanedione, pentanal, hexanal, 2-nonanone and (E,Z)-2,6-nonadienal. A similar trend was observed for pH 7-hydrolysate, for which the deaerator allowed a drop in the abundance of the seven following compounds (*p* < 0.05): 3-methylbutanal, 2-methylbutanal, 1-methyl-1H-tetrazole, pentanal, hexanal, (Z)-6-octen-2-one and 2-nonanone. It is of interest to note that, in this case, the deaerator conducted the total loss of 1-methyl-1H-tetrazole, pentanal and (Z)-6-octen-2-one. That could suggest that the ability of such device to remove volatile compounds was better at pH 7 than at pH 4. As it was mentioned previously, the hypothesis that looked more plausible to explain the difference in volatile compound content between the initial hydrolysate at different pH values was the following: while pH increased, higher interactions between the volatiles and other constituents occurred. This could be in line with the results of the deaerator for the hydrolysate at pH 4 and pH 7. Indeed, it seemed that this device could break weak interactions occurring between volatile compounds and other compounds present in the hydrolysate, resulting in a better decrease rate at pH 7 than at pH 4. Nevertheless, the deaerator did not lead to a decrease in the volatile content while the hydrolysate was treated at pH 10. This could indicate that, at pH 10, the chosen hypothesis was not enough to totally explain the mechanisms involved. It could be supposed that, at pH 10, a certain proportion of volatile compounds could take part in strong interactions, such as covalent bonds, but at the same time, some of them could have been lost due to their promoted passage in the headspace of the hydrolysate solution as well, or simply altered, hindering their detection. 

TMAO, TMA and DMA

The TMAO, TMA and DMA contents of HMH are shown in Table 8. Firstly, concerning the hydrolysate at the initial time for the three tested pH values, their concentration in TMAO, TMA and DMA was similar (*p* > 0.05). The only difference observed was related to the content of TMAO of the hydrolysate at pH 10, which was 20 times lower (*p* < 0.05) than those of the hydrolysate at pH 4 and 7 at the initial time. In this context, it is worth to mention that the procedures used for the analysis of TMAO, TMA and DMA recommend to alkalize samples of interest, to allow a better detection of these molecules based on their higher release into the sample headspace [41]. Therefore, the huge decrease in TMAO content observed in the hydrolysate at pH 10 could be related to a loss following their release into the headspace of the hydrolysate solution due to its high volatility.

Then, concerning the content in TMAO, TMA and DMA after the four ED treatments, no difference was observed between the hydrolysate at initial and final times. ED treatments were especially designed to assess whether TMA and DMA, two positively charged compounds at pH 4 and 7, were able to migrate. However, the results indicated that no migration happened while experiments were conducted with current. As suggested by the ED parameter analyses, some water dissociation took place during the treatments conducted at pH 4 and 7 with current. Therefore, it could be hypothesized that TMA and DMA had been in competition with the generated H^+^ to migrate into the CEMs, and that H^+^ could have prevailed over TMA and DMA. Another explanation could be that fouling occurring on CEMs, as suggested by the membrane conductivity analysis, hindering the migration of TMA and DMA, thus explaining such results. Chindapan et al. (2011) experienced, in their study, a decrease in TMA, and explained this result by its loss occurring during ED due to its high volatility [25]. However, the results obtained in the present study may indicate that TMA could not be lost as easily, since ED treatments carried out without current did not evidence any change in the content of this compound. Moreover, the fact that no change in the concentration of TMAO was observed between the hydrolysate at the initial and final times treated with current was more expected. Indeed, this molecule is a zwitterion, and the absence of global charge makes it less likely to migrate during an ED process.

The contents in TMAO, TMA and DMA of KCl recovery solution were analyzed (Table 9). The results show that the initial solution was free of these compounds, while the KCl solution at the final time of all the tested conditions only evidenced the presence of TMAO. The presence of TMAO in the recovery solution of treatments conducted with current was not expected, as the global charge of this compound was neutral. However, the fact that TMAO was present in the recovery solution of treatments carried out without current as well could suggest that another mechanism than electromigration could be involved. In addition, the concentration of TMAO in the different KCl recovery solutions was surprisingly as important as those of the corresponding hydrolysate at the initial time, and since the concentration of this compound in the hydrolysate did not evolve during the different ED treatments, this could suggest that new TMAO was generated over the time. The most logical explanation at the first glance could have been that some TMA evidenced oxidation, resulting in the formation of much more TMAO. However, this was not possible in the case of this study, as the initial hydrolysate, independently of its pH, had too low a content of TMA. This means that more complex mechanisms occurred. TMAO is traditionally produced from nitrogenous compounds, such as choline, betaine or carnitine, through metabolism pathways involving enzymes and gut microbiota [75]. Interestingly, HMH contains phospholipids whose choline can be a constituent and carnitine as well (Table 1). Even if metabolism pathways could not be involved in that case, it could be supposed that some TMAO was generated from the choline of phospholipids and carnitine through other reactions, such as oxidation. Nevertheless, this could only explain the occurrence of much more TMAO compared to the initial time, and not its recovery in the different KCl solutions. Another hypothesis could be that reactions between constituents of HMH, such as the choline of phospholipids or carnitine, as mentioned before, and those of CEMs could have taken place. This latter hypothesis seems to be even more plausible, as the analysis of ED parameters, and more specifically those regarding membrane conductivity, revealed that some interactions happened between hydrolysate constituents and membranes. However, at this stage, it is not possible to effectively favor one hypothesis rather than another one. A last point that is worth mentioning is that the absence of TMA and DMA in the KCl recovery solutions of treatments conducted with current was effectively representative of their non-migration.

Finally, the comparison of the performance of ED with those of a deaerator was assessed. The results are presented in Table 8. They indicate that the deaerator was only effective in decreasing the concentration of TMAO (*p* < 0.05) of the hydrolysate at pH 4 and 7. Regarding the hydrolysate at pH 10, this device had no effect on its composition. These results were consistent with those obtained for the most potent odor-active compounds, as discussed before. However, in that case, the fact that no impact regarding the TMAO content of the hydrolysate was observed gave credit to its loss following its release into the headspace of the hydrolysate sample, promoted by alkaline conditions and occurring before the deaerator treatment, as mentioned previously. 

## 4. Conclusions

GC-MS analysis allowed the identification of a total of 86 volatile compounds in the HMH. Among these 86, the following 15 were determined to be the most potent odor-active compounds of this hydrolysate by GC-O, combined with the detection frequency method: 3-methylbutanal, 2-methylbutanal, 1-methyl-1H-tetrazole, 2,3-pentanedione, pentanal, hexanal, (Z)-4-heptenal, heptanal, methional, benzaldehyde, (Z)-6-octen-2-one, (E,E)-2,4-heptadienal, octanal, 2-nonanone and (E,Z)-2,6-nonadienal. In addition, the HS-GC-NPD analysis revealed the presence of TMAO, TMA and DMA in the HMH. Furthermore, the performance of ED as a deodorization method was compared to that of a deaerator device. The results showed that pH had a huge impact on the volatile compound contents of the hydrolysate at the initial time. In fact, the abundance of the targeted molecules was lower at pH 10 than at pH 4, and intermediate at pH 7. While the pH increased from pH 4 to 7, volatile compounds were more involved in interactions with amino-acid-containing materials, explaining their lower availability and thus their lower abundance. However, at pH 10, more than one mechanism could be involved. Indeed, part of the targeted odor-active compounds should have been lost due to their volatility, while another part participated in irreversible bonds or was altered, hampering their detection. Regarding TMAO specifically, its lower content in the hydrolysate at pH 10 could be related to its loss resulting from a greater release into the headspace solution. On the other hand, ED did not affect the volatile compound contents of HMH. Concerning ED treatments conducted with current, no migration of volatile compounds, and more precisely no migration of TMA and DMA, occurred. Two phenomena were considered to be the main possible limiting process conditions regarding the removal of the targeted compounds. The first was the occurrence of fouling on IEM due to both electrostatic and hydrophobic interactions between IEM and HMH constituents, including volatile compounds. The second was the occurrence of water dissociation on the IEM interfaces due to the reaching of LCD, as well as the presence of water dissociation catalyzers involved in both IEM and HMH constituents. Moreover, the fact that ED treatments without current did not impact the volatile compound contents implied that no loss of these molecules due to their volatile nature happened during the circulation of the hydrolysate solution in an ED system. Interestingly, independently of the pH and current conditions of the ED treatments, it appeared that new TMAO was generated over the time. Two hypotheses were considered. The first would be that TMAO was generated directly in the hydrolysate solution from its precursors, while the second could involve its precursors, as well as the constituents of CEMs. On the contrary to ED, treatments conducted by deaerator significantly decreased the abundance of the targeted compounds at pH 4 and 7, but had no effect at pH 10. Therefore, the conditions leading to the best removal levels of the targeted volatile compounds were the deaerator treatment performed on the hydrolysate at pH 7, and the alkalization of this latter until pH 10. Despite the fact that the relevance of ED to be used as a deodorization method of HMH was not proven at that stage, it appeared that the establishment of strategies to avoid both fouling and water dissociation phenomena could lead to a better process efficiency. However, this supposes to deepen the knowledge regarding, especially, the fouling resulting from interactions between IEM and HMH constituents, with particular interest in those involving volatile compounds, by performing Attenuated Total Reflection–Fourier Transform Infrared (ATR-FTIR), as well as identifying the mechanisms leading to TMAO formation during ED. This is currently under investigation. Finally, the use of electromembrane processes, other than conventional electrodialysis, could be another promising solution that is worth further investigation. Therefore, electromembrane processes have a chance to become an effective deodorization method in the future.

## Figures and Tables

**Table 1:** Chemical composition of the herring milt hydrolysate (HMH) according to the manufacturer specifications.

Compounds	Composition of Dry Powder (%)
Proteins	40.0 ± 5.0
Arginine	16.0 ± 2.0
Leucine	2.0 ± 0.3
Lysine	1.8 ± 0.3
Isoleucine	1.1 ± 0.2
Histidine	0.4 ± 0.2
Methionine	<0.1
Nucleic acids	26.0 ± 5.0
Lipids	14.0 ± 4.0
Phospholipids	4.5 ± 1.5
Omega-3 (n-3) acids: Eicosapentaenoic acid (EPA) and docosahexaenoic acid (DHA)	4.0 ± 1.0
Minerals	12.5 ± 2.5
Phosphorus	3.2 ± 0.6
Sodium	1.2 ± 0.1
Potassium	0.7 ± 0.1

**Table 2:** Volatile compounds identified in HMH.

Classification	Compounds	Chemical Formula	Retention Time (Min)
**Aldehydes**	Butanal	C_4_H_8_O	6.050
	(E)-2-Butenal	C_4_H_6_O	7.086
	3-Methylbutanal	C_5_H_10_O	7.200
	2-Methylbutanal	C_5_H_10_O	7.381
	Pentanal	C_5_H_10_O	8.009
	2-Methyl-2-pentenal	C_6_H_10_O	8.777
	(E)-2-Methyl-2-butenal	C_5_H_8_O	8.968
	(E)-2-Pentenal	C_5_H_8_O	9.201
	Hexanal	C_6_H_12_O	10.121
	2-Ethyl-trans-2-butenal	C_6_H_10_O	10.786
	Furfural	C_5_H_4_O_2_	10.845
	2-Hexenal	C_6_H_10_O	11.235
	2-Ethyl-2-pentenal	C_7_H_12_O	11.669
	(Z)-4-Heptenal	C_7_H_12_O	12.114
	Heptanal	C_7_H_14_O	12.156
	Methional	C_4_H_8_OS	12.338
	(E,E)-2,4-Hexadienal	C_6_H_8_O	12.391
	Benzaldehyde	C_7_H_6_O	13.552
	(E,E)-2,4-Heptadienal	C_7_H_10_O	13.978
	Benzeneacetaldehyde	C_8_H_8_O	14.984
	4-(1-methylethyl)-1-cyclohexene-1-carboxaldehyde	C_10_H_16_O	16.025
	2-Isopropyl-5-oxohexanal	C_9_H_16_O_2_	16.200
	(E,Z)-2,6-Nonadienal	C_9_H_14_O	16.642
	2-Phenylpropenal	C_9_H_8_O	18.887
	4-Ethyl-benzaldehyde	C_9_H_10_O	17.027
	Decanal	C_10_H_20_O	17.411
	Lilac aldehyde D	C_10_H_16_O_2_	19.394
**Ketones**	2,3-Pentanedione	C_5_H_8_O_2_	7.918
	2,3-Hexanedione	C_6_H_10_O_2_	9.751
	2-Hexanone	C_6_H_12_O	9.854
	2-Heptanone	C_7_H_14_O	11.887
	6-Methyl-2-heptanone	C_8_H_16_O	13.115
	(Z)-6-Octen-2-one	C_8_H_14_O	13.680
	2-Octanone	C_8_H_16_O	13.772
	5-Methyl-3-hepten-2-one	C_8_H_14_O	13.873
	3,5-Octadien-2-one	C_8_H_12_O	15.215
	Acetophenone	C_8_H_8_O	15.396
	2-Nonanone	C_9_H_18_O	15.526
	(E,E)-3,5-octadien-2-one	C_8_H_12_O	15.650
	2-Decanone	C_10_H_20_O	17.155
	2-(2-nitro-2-propenyl)-cyclohexanone	C_9_H_13_NO_3_	17.890
	2-Undecanone	C_11_H_22_O	18.679
	7-Methylene-6 (or 8)-methyl-bicyclo [3.3.0]octan-2-one	C_10_H_14_O	18.918
	2,6-Di-tert-butyl-4-hydroxy-4-methylcyclohexa-2,5-dien-1-one	C_15_H_24_O_2_	21.084
**Alcohols**	2-Methylbutan-2-ol	C_5_H_12_O	6.882
	1-Penten-3-ol	C_5_H_10_O	7.682
	1-Pentanol	C_5_H_12_O	9.358
	(Z)-2-Penten-1-ol	C_5_H_10_O	9.412
	1-Hexen-3-ol	C_6_H_12_O	9.649
	(Z)-3-Octen-2-ol	C_8_H_16_O	10.494
	2-Octyn-1-ol	C_8_H_14_O	13.361
	(E)-2-Octen-1-ol	C_8_H_16_O	14.045
	2,7-Octadien-1-ol	C_8_H_14_O	15.170
	6,6-Dimethyl-bicyclo[3 .1.1]hept-2-ene-2-methanol	C_10_H_16_O	17.641
	5-(methylenecyclopropyl)-1-pentanol	C_9_H_16_O	19.758
	2,6-bis(1,1-dimethylethyl)-1,4-benzenediol	C_14_H_22_O_2_	21.317
**Alkenes**	2,3-Dimethyl-2-butene	C_6_H_12_	6.227
	(E)-3-Methyl-2-pentene	C_6_H_12_	6.471
	1,4-Cyclohexadiene	C_6_H_8_	8.307
	Toluene	C_7_H_8_	9.573
	(Z,Z)-3,5-Octadiene	C_8_H_14_	10.337
	3,5,5-Trimethyl-2-hexene	C_9_H_18_	13.502
	1,3-bis(1,1-dimethylethyl)-benzene	C_14_H_22_	18.192
	Butylated Hydroxytoluene	C_15_H_24_O	21.730
**Nitrogenous** **compounds**	Dimethylamine *	C_2_H_7_N	4.350
	Trimethylamine *	C_3_H_9_N	4.581
	Trimethylamine oxide *	C_3_H_9_NO	4.883
	1-Methyl-1H-tetrazole	C_2_H_4_N_4_	7.739
	3-Methyl-butanenitrile	C_5_H_9_N	8.716
	N-Nitrosodimethylamine	C_2_H_6_N_2_O	8.862
	Methylpyrazine	C_5_H_6_N_2_	10.696
	3-Methyl oxime butanal	C_5_H_11_NO	10.992
	2,5-Dimethylpyrazine	C_6_H_8_N_2_	12.449
	2-Acetylthiazole	C_5_H_5_NOS	14.513
	Benzothiazole	C_7_H_5_NS	18.255
**Alkanes**	Cyclohexane	C_6_H_12_	6.704
	Hexylidene cyclopropane	C_9_H_16_	9.912
	4-Methyloctane	C_9_H_20_	11.355
	Dodecane	C_12_H_26_	14.903
	(1.alpha.,3.alpha.,5.alpha.)-1,5-diethenyl-3-methyl-2-methylene-cyclohexane	C_12_H_18_	20.135
**Furans**	2-Methylfuran	C_5_H_6_O	9.155
	5-Isopropyl-3,3-dimethyl-2-methylene-2,3-dihydrofuran	C_10_H_16_O	16.740
**Esters**	Cis-cyclohexane-1,4-dimethanol diacetate	C_12_H_20_O_4_	18.508
	2,2,4-trimethyl-1,3-pentanediol diisobutyrate	C_16_H_30_O_4_	22.692
**Others**	Trimethyloxirane	C_5_H_10_O	6.5140
	Di-t-butylacetylene	C_10_H_18_	12.546
	(Z)-3-undecen-1-yne	C_11_H_18_	15.796
	2-(hexyn-1-yl)-3-methoxymethylene oxirane	C_10_H_14_O_2_	18.042
	Acetic acid, nonyl ester	C_11_H_22_O_2_	18.820

* Compounds identified by HS-GC-NPD while all the others were identified by GC-MS.

**Table 3:** Most potent odor-active volatile compounds of HMH.

RT (Min)	Compounds	No. of Judges ^a^	Average Intensity ^b^	No. of Judges Having Qualified the Odor as Bad or Good ^c^		Odorant Properties ^d^	
				Bad	Good	Descriptors	Odor Threshold (µg/kg)
7.200	3-Methylbutanal	13	4.0	5	6	Burnt, malted, dark chocolate	0.2–2.0
7.381	2-Methylbutanal	6	3.0	1	4	Burnt, malted, dark chocolate	1.0–3.0
7.739	1-Methyl-1H-tetrazole	9	2.0	5	3	-	-
7.918	2,3-Pentanedione	12	3.0	0	10	Butter, fruity	15–5505.6
8.009	Pentanal	7	2.0	3	4	Pungent	12–42
10.121	Hexanal	10	2.0	1	7	Green	5.0
12.114	(Z)-4-heptenal	7	3.0	5	2	Fishy	0.04–0.8
12.156	Heptanal	6	3.0	3	2	Green, rancid, fishy	0.7–2.9
12.338	Methional	11	3.0	5	5	Cooked potatoes	0.2
13.552	Benzaldehyde	13	3.0	9	3	Almond, nutty, fruity	41.7–4600
13.680	(Z)-6-octen-2-one	9	3.0	7	0
13.978	(E,E)-2,4-heptadienal	5	2.0	3	2	Fatty, fishy	10–15.4
14.045	Octanal	7	3.0	1	6	Citrus, orange, fatty, fishy	0.6
15.526	2-Nonanone	12	4.0	8	3	Grass, green	200
16.642	(E,Z)-2,6-nonadienal	12	3.0	4	6	Cucumber-like	1

^a^ Number of judges (out of thirteen) who have perceived an odor. ^b^ The average intensity given by the judges (out of thirteen) who perceived an odor. The average intensity was rounded to the nearest whole number i.e., an average intensity between 2.0 and 2.5 was rounded to 2.0, and an average intensity between 2.5 and 3.0 was rounded to 3.0. ^c^ The judges were asked to qualify the odor as bad or good. Sometimes the total number was inferior to those related to the number of judges having perceived an odor, since some of them simply considered the odor to be OK. ^d^ Odorant properties were indicated only when they were available. They were gathered from the following literature and online database [9,11,16,21,33,34,35,36,45,46,47,54,55]; (http://www.odour.org.uk; http://www.flavornet.org).

**Table 4:** Ash content of the HMH at the initial time and after the different ED treatments (mean ± standard deviation).

	pH 4			pH 7		
	**Initial Time**	**Final Time (ED + Current)**	**Final Time (ED No Current)**	**Initial Time**	**Final Time (ED + Current)**	**Final Time (ED No Current)**
**Ash content (%)**	0.478 ± 0.005^ a^	0.400 ± 0.018^ c^	0.460 ± 0.020^ ab^	0.484 ± 0.007^ a^	0.427 ± 0.020^ bc^	0.462 ± 0.017^ ab^

Values within the same row with different letters (a–c) are significantly different *p* < 0.05 (Tukey test).

**Table 5:** Ash content of the KCl recovery solution at initial time and after the different ED treatments (mean ± standard deviation).

		pH 4		pH 7	
	**Initial Time**	**Final Time (ED + Current)**	**Final Time (ED No Current)**	**Final Time (ED + Current)**	**Final Time (ED No Current)**
**Ash content (%)**	0.166 ± 0.007^ b^	0.240 ± 0.022^ a^	0.140 ± 0.023^ b^	0.230 ± 0.005^ a^	0.167 ± 0.016^ b^

Values within the same row with different letters (a–b) are significantly different, *p* < 0.05 (Tukey test).

**Table 6:** Abundance of the most-potent odor active compounds of HMH (×10^7^ Arbitrary Unit (A.U)) at the initial time and after the different treatments (mean ± standard deviation).

	pH 4				pH 7				pH 10	
	**Initial Time**	**Final Time (ED + Current)**	**Final Time (ED No Current)**	**Final Time Deaerator**	**Initial Time**	**Final Time (ED + Current)**	**Final Time (ED No Current)**	**Final Time Deaerator**	**Initial Time**	**Final Time Deaerator**
3-Methylbutanal	10.3 ± 2.14 ^a^	7.61 ± 0.52 ^ab^	7.67 ± 1.00 ^ab^	3.36 ± 0.15 ^cd^	6.47 ± 1.61 ^b^	5.53 ± 0.40 ^bc^	4.89 ± 0.50 ^bc^	1.26 ± 0.05 ^d^	4.88 ± 0.12 ^bc^	5.08 ± 0.80 ^bc^
2-Methylbutanal	4.15 ± 2.06 ^a^	3.87 ± 0.46 ^a^	3.32 ± 0.69 ^ab^	1.00 ± 0.05 ^bc^	3.67 ± 1.03 ^a^	2.66 ± 0.54 ^abc^	2.42 ± 0.55 ^abc^	0.831 ± 0.094 ^c^	2.45 ± 0.14 ^abc^	2.13 ± 0.61 ^abc^
1-Methyl-1H-tetrazole	0.00 ± 0.00^b^	0.00 ± 0.00 ^b^	0.00 ± 0.00 ^b^	0.00 ± 0.00 ^b^	3.80 ± 0.92 ^a^	3.94 ± 0.55 ^a^	2.95 ± 0.73 ^a^	0.00 ± 0.00 ^b^	0.00 ± 0.00 ^b^	0.00 ± 0.00 ^b^
2,3-Pentanedione	8.90 ± 1.66 ^a^	7.03 ± 0.63 ^ab^	7.13 ± 0.79 ^ab^	5.02 ± 0.72 ^bc^	3.05 ± 0.94 ^cd^	2.94 ± 0.22 ^cd^	2.53 ± 0.22 ^d^	1.60 ± 0.19 ^de^	0.356 ± 0.072 ^e^	0.237 ± 0.018 ^e^
Pentanal	3.76 ± 0.10 ^a^	3.35 ± 0.28 ^ab^	3.64 ± 0.23 ^a^	1.31 ± 0.02 ^cd^	2.79 ± 1.75 ^abc^	3.37 ± 0.40 ^ab^	2.77 ± 0.40 ^abc^	0.00 ± 0.00 ^d^	1.67 ± 0.11 ^bcd^	1.18 ± 0.14 ^cd^
Hexanal	6.94 ± 1.54 ^a^	4.63 ± 1.88 ^abc^	5.85 ± 0.90 ^ab^	3.66 ± 0.08 ^bc^	5.30 ± 1.34 ^ab^	4.69 ± 0.27 ^abc^	3.78 ± 0.66 ^bc^	2.47 ± 0.15 ^c^	2.14 ± 0.27 ^c^	2.18 ± 0.55 ^c^
(Z)-4-heptenal	4.66 ± 2.59 ^a^	4.40 ± 0.32 ^a^	4.75 ± 0.94 ^a^	4.11 ± 0.26 ^ab^	3.19 ± 1.25 ^abc^	2.37 ± 0.23 ^abc^	2.20 ± 0.13 ^abc^	1.48 ± 0.19 ^bc^	1.13 ± 0.05 ^bc^	0.888 ± 0.166 ^bc^
Heptanal	2.21 ± 0.97 ^ab^	1.81 ± 0.11 ^abc^	2.29 ± 0.57 ^a^	1.42 ± 0.18 ^abcd^	1.63 ± 0.80 ^abcd^	1.16 ± 0.16 ^abcd^	1.10 ± 0.36 ^abcd^	0.909 ± 0.041 ^bcd^	0.582 ± 0.107 ^cd^	0.358 ± 0.112 ^d^
Methional	0.33 ± 0.19 ^a^	0.267 ± 0.064 ^ab^	0.277 ± 0.056 ^ab^	0.215 ± 0.042 ^abc^	0.084 ± 0.055 ^bcd^	0.060 ± 0.015 ^cd^	0.046 ± 0.027 ^cd^	0.053 ± 0.005 ^cd^	0.013 ± 0.004 ^cd^	0.00 ± 0.00 ^d^
Benzaldehyde	5.34 ± 2.48 ^a^	5.67 ± 1.93 ^a^	4.80 ± 1.74 ^a^	6.46 ± 0.56 ^a^	5.19 ± 1.25 ^a^	5.10 ± 0.81 ^a^	4.12 ± 0.96 ^a^	2.72 ± 0.28 ^a^	3.49 ± 0.73 ^a^	3.36 ± 0.31 ^a^
(Z)-6-octen-2-one	1.07 ± 0.44 ^a^	0.414 ± 0.273 ^bc^	0.720 ± 0.150 ^ab^	0.465 ± 0.115 ^abc^	0.684 ± 0.277 ^ab^	0.591 ± 0.299 ^abc^	0.683 ± 0.093 ^ab^	0.00 ± 0.00 ^c^	0.891 ± 0.093 ^ab^	0.275 ± 0.080 ^bc^
(E,E)-2,4-heptadienal	9.84 ± 1.88 ^a^	6.11 ± 0.79 ^b^	7.15 ± 2.01 ^ab^	9.05 ± 0.62 ^a^	0.879 ± 0.326 ^c^	0.770 ± 0.225 ^c^	0.596 ± 0.247 ^c^	1.31 ± 0.12 ^c^	0.306 ± 0.097 ^c^	0.225 ± 0.079 ^c^
Octanal	2.73 ± 0.93 ^a^	1.67 ± 0.17 ^abc^	1.64 ± 0.67 ^abc^	1.67 ± 0.08 ^abc^	1.88 ± 0.71 ^ab^	1.14 ± 0.16 ^bcd^	1.25 ± 0.41^bcd^	1.04 ± 0.09 ^bcd^	0.428 ± 0.016 ^cd^	0.294 ± 0.033 ^d^
2-Nonanone	1.38 ± 0.47 ^a^	1.04 ± 0.29 ^ab^	1.14 ± 0.30 ^ab^	0.578 ± 0.015 ^bc^	1.41 ± 0.56 ^a^	0.694 ± 0.140 ^abc^	0.757 ± 0.158 ^abc^	0.236 ± 0.041 ^c^	0.569 ± 0.062 ^bc^	0.149 ± 0.037 ^c^
(E,Z)-2,6-nonadienal	3.72 ± 0.97 ^a^	2.24 ± 0.13 ^b^	2.54 ± 0.78 ^ab^	1.36 ± 0.17 ^bc^	0.699 ± 0.294 ^cd^	0.580 ± 0.073 ^cd^	0.523 ± 0.044 ^cd^	0.461 ± 0.044 ^cd^	0.030 ± 0.006 ^d^	0.030 ± 0.009 ^d^

Values within the same row with different letters (a–e) are significantly different *p* < 0.05 (Tukey test).

**Table 7:** Abundance of the most-potent odor active compounds of HMH recovered in the KCl solution (×10^7^ A.U) after the different ED treatments (mean ± standard deviation).

		pH 4		pH 7	
	**Initial Time**	**Final Time (ED + Current)**	**Final Time (ED No Current)**	**Final Time (ED + Current)**	**Final Time (ED No Current**)
3-Methylbutanal	0.00 ± 0.00 ^a^	0.027 ± 0.143 ^a^	0.133 ± 0.030 ^a^	0.960 ± 1.318 ^a^	0.097 ± 0.151 ^a^
Hexanal	0.00 ± 0.00 ^b^	2.19 ± 0.47 ^a^	1.71 ± 0.70 ^a^	2.33 ± 0.17 ^a^	1.86 ± 0.12 ^a^
Heptanal	0.00 ± 0.00 ^a^	0.156 ± 0.188 ^a^	0.025 ± 0.082 ^a^	0.026 ± 0.091 ^a^	0.095 ± 0.138 ^a^
Benzaldehyde	0.00 ± 0.00 ^c^	4.54 ± 0.46 ^a^	0.731 ± 0.019 ^b^	0.595 ± 0.090 ^b^	0.684 ± 0.042 ^b^
Octanal	0.00 ± 0.00 ^b^	0.202 ± 0.089 ^ab^	0.058 ± 0.055 ^ab^	0.214 ± 0.143 ^ab^	0.333 ± 0.182 ^a^
2-Nonanone	0.00 ± 0.00 ^c^	0.026 ± 0.00 ^a^	0.017 ± 0.003 ^ab^	0.003 ± 0.012 ^bc^	0.003 ± 0.005 ^bc^

Values within the same row with different letters (a–c) are significantly different, *p* < 0.05 (Tukey test).

**Table 8:** TMA, DMA and TMAO content of HMH at the initial time and after the different treatments (mean ± standard deviation).

	pH 4				pH 7				pH 10	
	**Initial Time**	**Final Time (ED + Current)**	**Final Time (ED No Current)**	**Final Time Ddeaerator**	**Initial Time**	**Final Time (ED + Current)**	**Final Time (ED No Current)**	**Final Time Ddeaerator**	**Initial Time**	**Final Time Ddeaerator**
TMA (ppm)	3.55 ± 0.60 ^abc^	3.08 ± 0.99 ^abcd^	2.39 ± 2.05 ^cd^	3.67 ± 0.37 ^abc^	4.88 ± 0.27 ^abc^	5.54 ± 0.59 ^a^	4.95 ± 1.01 ^ab^	2.50 ± 0.61 ^bcd^	2.63 ± 0.20 ^bcd^	0.72 ± 0.04 ^d^
DMA (ppm)	5.31 ± 0.33 ^a^	4.58 ± 2.04 ^a^	4.12 ± 3.57 ^a^	5.63 ± 1.05 ^a^	2.54 ± 0.25 ^a^	2.76 ± 0.50 ^a^	2.51 ± 0.19 ^a^	3.02 ± 0.31 ^a^	4.26 ± 0.11 ^a^	3.78 ± 0.09 ^a^
TMAO (ppm)	942.36 ± 135.9 ^a^	957.00 ± 153.79 ^a^	1019.29 ± 155.12 ^a^	155.63 ± 46.51 ^b^	1023.67 ± 63.10 ^a^	1004.00 ±31.24 ^a^	948.83 ± 75.03 ^a^	121.91 ± 44.55 ^b^	52.87 ± 15.03 ^b^	101.37 ± 26.05 ^b^

Values within the same row with different letters (a–d) are significantly different *p* < 0.05 (Tukey test).

**Table 9:** TMA, DMA and TMAO content of KCl recovery solution at initial time and after the different ED treatments (mean ± standard deviation).

		pH 4		pH 7	
	**Initial Time**	**Final Time (ED + Current)**	**Final Time (ED No Current)**	**Final Time (ED + Current)**	**Final Time (ED No Current)**
TMA (ppm)	<0.02 ^a^	<0.02 ^a^	<0.02 ^a^	<0.02 ^a^	<0.02 ^a^
DMA (ppm)	<1.00 ^a^	<1.00 ^a^	<1.00 ^a^	<1.00 ^a^	<1.00 ^a^
TMAO (ppm)	<2.50 ^b^	1083.64 ± 158.89 ^a^	967.67 ± 127.79 ^a^	1026.73 ± 143.80 ^a^	1013.88 ± 142.96 ^a^

Values within the same row with different letters (a–b) are significantly different *p* < 0.05 (Tukey test).
